# A planar tracking strategy based on multiple-interpretable improved PPO algorithm with few-shot technique

**DOI:** 10.1038/s41598-024-54268-6

**Published:** 2024-02-16

**Authors:** Xiao Wang, Zhe Ma, Lu Cao, Dechao Ran, Mingjiang Ji, Kewu Sun, Yuying Han, Jiake Li

**Affiliations:** 1 College of Information Science and Technology, Beijing University of Chemical Technology, Beijing, 100029 China; 2 Intelligent Science & Technology, Academy Limited of CASIC, Beijing, 100043 China; 3 Key Lab of Aerospace Defense Intelligent System and Technology, Beijing, 100043 China; 4 National Innovation Institute of Defense Technology, Academy of Military Sciences, Beijing, 100071 China

**Keywords:** Tracking problem, Reinforcement learning, Interpretable Learning, Quantum computation, Electrical and electronic engineering, Mechanical engineering

## Abstract

Facing to a planar tracking problem, a multiple-interpretable improved Proximal Policy Optimization (PPO) algorithm with few-shot technique is proposed, namely F-GBQ-PPO. Compared with the normal PPO, the main improvements of F-GBQ-PPO are to increase the interpretability, and reduce the consumption for real interaction samples. Considering to increase incomprehensibility of a tracking policy, three levels of interpretabilities has been studied, including the perceptual, logical and mathematical interpretabilities. Detailly speaking, it is realized through introducing a guided policy based on Apollonius circle, a hybrid exploration policy based on biological motions, and the update of external parameters based on quantum genetic algorithm. Besides, to deal with the potential lack of real interaction samples in real applications, a few-shot technique is contained in the algorithm, which mainly generate fake samples through a multi-dimension Gaussian process. By mixing fake samples with real ones in a certain proportion, the demand for real samples can be reduced.

## Introduction

Reinforcement learning (RL) is a way to optimize strategies by maximizing returned reward through interaction with the environment. It makes optimization problems only related to state-action pairs, and thus becomes an important technical approach to solve complex decision-making problems.

Since the 1980s, single-agent RL can be approximately classified into two types, including data-driven RL and knowledge-driven RL^1^. For data-driven type, the development can be divided into the following stages. (1) The tabular reinforcement learning in discrete-state space, which is mainly represented by Q-learning or SARSA-learning. It is successfully applied in finite-discrete space with the help of time-difference technique^2^. (2) The deep-Q-network (DQN) reinforcement learning^3^, where the value function is represented as a parameterized network, thus solving the dimensional disaster^2^. Besides, various variants (such as Nature DQN and Double DQN) have been gradually proposed^4,5^ after that. (3) The kind of Actor-Critic reinforcement learning, which simultaneously parameterizes value function and policy function. Benefiting from the latest research on deep neural networks and the advantages of multi-architecture optimization algorithms^6–8^, some optimized versions have been attempted^9,10^. Represented by “Deepmind” from Google, with the in-depth discussion of experience-replay, exquisite design of advantage function, and the attention mechanism, deep RL is still making breakthroughs^11–15^. In addition to field of games represented by Alpha Go and Starcraft^16^, data-driven RL can also be employed in the agents that have real physical meanings. Combined with fuzzy inference rules and neural networks, RL has been applied to the trajectory tracking, the tuning of control parameter, as well as trajectory planning for robots^17–19^. Besides, RL has shown different degrees of application potential in ground attack-defense problem, defending of specific blocks, and anti-collision problems^20–22^. In addition, in terms of multi-system cooperation, self-organizing flight based on RL has also made breakthroughs in UAV swarm^23,24^ and space cluster^25,26^. However, the agents that have real physical meanings are often characterized by high interaction costs, few sample sources and high maintenance costs^27^. To deal with these agents, a type of model-driven reinforcement learning is developed. The main concept is to first obtain the model of the environment where the agent resides and then simulate the sequential decision of tasks from the model. At present, the model-based RL can be generally divided into the four main types. (1) The method of model fitting and optimal control, which is represented by Probabilistic Ensembles with Trajectory Sampling (PEST)^28^. Through skipping the process of gradient calculation, the optimal policy will be directly found by the forward propagation based on a series of trainable Gaussian models. Using the idea of model predictive control, the optimal action sequence can be planned. (2) The method of model fitting and policy episode, which is adopted by the algorithms based on Dyna framework. The typical examples are the methods of Model-Based Value Expansion method^29^ and Stochastic Ensemble Value Expansion^30^, which can balance the proportion of model-based and model-free parts through a H-step rollout. (3) The method of theoretical analysis, which is represented by Stochastic Lower Bound Optimization. The policy is expected to be monotonically improved based on the deduction of the close degree between the training and real polices. On this basis, the method of Model-Based Policy Optimization^31^ has been also proposed, which further simplifies and updates the monotonicity conditions. (4) "White box" method, which is represented by Probabilistic inference for learning control (PILCO)^32^. Using the idea of Gaussian process regression, the model of probabilistic dynamics can be conducted.

To sum up, the theoretical research on RL has been developed rapidly in recent years. However, it is found that there are some problems in the actual application, such as inconformity with human intuition, difficulty in tracing the results and incomprehensibility of the model. These problems can be summarized as the lack of interpretability of the algorithm. (It is hard to absolutely distinguish the roles of “interpretability” and “explainability” at present, but guided by^33^, we uniformly call it as “interpretability”, since the passive characteristics of the algorithm is more concerned about in this paper). In fact, not just RL, other artificial intelligence techniques are experiencing the same difficulty to a great extent. The interpretability is to enable human users to understand and trust the results or outputs produced by those learning algorithms. If an algorithm has a good interpretability, it will help to describe the model accuracy, calculation fairness and result credibility in an intelligent decision-making process.

At present, the current definition of "interpretability" can be explained from different views. In a metaphysical concept, it is suggested that interpretability should be defined based on abstract words, such as credibility and reliability^32^. However, in a physical concept, it is advocated to define it based on the practical significance of interpretation from the perspectives of philosophy and mathematics^34–36^. If the models cannot be understood by human beings, they certainly cannot be effectively evaluated^37^. Therefore, the basic principle of an interpretable learning algorithm should be similar to that of human learning cognition. In this way, corresponding to the detailed design of specific algorithms, the interpretability can be realized from three level. (1) Perceptual interpretability: The rationality of decisions can be intuitively understood without the knowledge from any specific domain; (2) Logical interpretability: The main logic of the algorithm is reasonable, and it can make sense when checked by human experts; (3) Mathematical interpretability: The framework design of the algorithm should be accurate and reasonable, having the ability to integrate into the current knowledge system^38^.

Planar tracking problem is a typical kind of differential game. In real application, the problems of the air combat and the ground round-up are continually attracting the researcher’s attentions. The most conventional scenario in a tracking problem is a PE game, where there exits one pursuer and one evader. It is promising that the game policy will be improved to be more powerful if one can design an advanced training framework which is interpretable and explains well to human. Therefore, facing to this typical planar tracking problem, this paper proposes a multiple-interpretable improved Proximal Policy Optimization (PPO) algorithm with few-shot technique in view of the lack of interpretability of RL in the past research. Specifically speaking, the algorithm is designed from three interpretable levels including perception, logic and mathematics. Besides, considering to save the consumption of real interaction samples, a few-shot technique based on multi-dimension Gaussian process is studied. The key contributions of this paper are listed as follows:

1. For the perceptual interpretability, a guided policy based on Apollonius circle is added to assist decision-making. Since Apollonius circle is with the theoretical guarantee, it has a good interpretability that one can easily verify it through algebraic calculation and judge its correctness from a perceptual view. Therefore, by introducing this guided policy, the perceptual interpretability of the algorithm can be effectively improved, and the rationality of the decision can be more intuitively understanded.
2. For the logical interpretability, the forms of new and old policies in typical PPO2 are redesigned. By imitating the Levy and Brown motions in nature, the exploration policy for the agent is reconstructed based on the hybrid biological motion laws. Benefited from the inherent rationality of the natural exploration, the execution logic of algorithm is endowed with interpretability.
3. For the mathematical interpretability, a loop of external parameter update is added. Due to the introductions of guided policy and biological exploration policy, some external parameters appear. Based on the quantum genetic algorithm (QGA), those parameters can be optimized, building up an extra loop except for the update of internal policy network. Employing the quantum bit, the external parameters can be coded in a quantum chromosome and optimized by the QGA. Since QGA has the characteristics of evolutionary methods, the proposed algorithm will be mathematically interpretable.
4. Considering to reduce the real sample usage for the agent, a few-shot technique is also attempted. Based on the storage of the previous real samples, the future states can be predicted to form fake samples through multi-dimensional Gaussian process. In this way, the consumption of real samples can be saved to a certain extent. Different from other few-shot methods (such as GAN and meta-learning ways), the technique used here does not need training and the generation of fake samples can be obtained quickly.

The rest structure of this paper is as follows: The second chapter introduces the main concept of PPO, biological motion laws and basis of quantum computation, which are the necessary preliminaries involved in this paper; the third chapter illustrates the overall design of the proposed few-shot multiple-interpretable improved PPO; the fourth chapter detailly introduces the improvements of multiple-interpretable algorithm including the guided policy, biological exploration policy, and the external parameter updates, as well as the few-shot technique; the fifth chapter analyzes the performance of the proposed algorithm through numerical simulation, and the last chapter is the summary of the full text.

## Preliminaries

The proposed algorithm can be seen as an improved version of PPO; therefore, this chapter will introduce the main concept of PPO. In addition, since the two motion laws in nature and the quantum computation will be involved, these contents will also be briefly described in the following.

### Proximal policy optimization

For a general reinforcement learning goal, the objective function $g$ for the agent can be described as

1 $$\widehat{g}={\widehat{E}}_{t}\left[{\nabla }_{\theta }\mathit{log}{\pi }_{\theta }\left({a}_{t}|{s}_{t}\right){\widehat{A}}_{t}\right]$$

where ${\pi }_{\theta }$ is a stochastic policy function, ${\widehat{A}}_{t}$ is the estimated value of the advantage function at time $t$, ${s}_{t}$ is the current state and ${a}_{t}$ is the current action. For an on-policy algorithm, the policy used in the sampling and the one used in the update is the same one. In such a way, the efficiency is relatively low, since the samples can only be used once for updating. Therefore, to make the algorithm “off-policy”, the technique of important sampling should be employed. Suppose there are policies $p$ and $q$ for the agent, an objective function $f(x)$ which the agent needs to optimize can be expressed as

2 $$\begin{array}{c}{E}_{x\sim p}[f(x)]\approx \frac{1}{N}{\sum }_{i=1}^{N}f({x}_{i})=\int f(x)p(x)dx\\ =\int f(x)\frac{p(x)}{q(x)}q(x)dx={E}_{x\sim q}[f(x)\frac{p(x)}{q(x)}]\end{array}$$

where $N$ represents the sampling times. From left side of Eq. (2), it is seen that the agent needs to improve policy $p$ with the relative samples under policy $p$. In this way, it is on-policy. However, after the transformation, the samples obtained from policy $q$ can help to update policy $p$, which is shown in the right ride of Eq. (2). Certainly, only if the distributions of $p$ and $q$ are similar, the equation can be formed; otherwise, there may exist heavy difference between ${E}_{x\sim p}[f(x)]$ and ${E}_{x\sim q}[f(x)\frac{p(x)}{q(x)}]$. Benefited from important sampling, the policy update can be changed from “on-policy” way to “off-policy” way, and this is the core idea of PPO. Two different policies are need, and denoted as $\pi$ and $\pi {\prime}$, where $\pi$ is used to interact with the environment and $\pi {\prime}$ is the one that need to be updated. To make sure the difference between $\pi$ and $\pi {\prime}$ not differ much, a divergence $KL\left[\pi \left(\cdot |{s}_{t}\right),{\pi }^{\mathrm{^{\prime}}}\left(\cdot |{s}_{t}\right)\right]$ is used, and learning goal can be rewritten as below.

3 $${max}_{\theta }{\widehat{E}}_{t}\left[\frac{{\pi }^{\mathrm{^{\prime}}}\left({a}_{t}|{s}_{t}\right)}{\pi \left({a}_{t}|{s}_{t}\right)}{A}_{t}^{\mathrm{^{\prime}}}-\beta KL\left[\pi \left(\cdot |{s}_{t}\right),{\pi }^{\mathrm{^{\prime}}}\left(\cdot |{s}_{t}\right)\right]\right]$$

On the basis, a more beneficial version is proposed and called as PPO2. In PPO2, the loss function can be designed as

4 $${L}^{Clip}\left(\theta \right)={\widehat{E}}_{t}\left[\mathit{min}\left({r}_{t}\left(\theta \right){\widehat{A}}_{t},clip\left({r}_{t}\left(\theta \right),1-\varepsilon ,1+\varepsilon \right){\widehat{A}}_{t}\right)\right]$$

where ${r}_{t}\left(\theta \right)=\frac{{\pi }^{\mathrm{^{\prime}}}\left({a}_{t}|{s}_{t}\right)}{\pi \left({a}_{t}|{s}_{t}\right)}$ and $\varepsilon$ is a hyper parameter. The main advance in this loss function is to use a clip function. When ${\widehat{A}}_{t}>0$, it means that the current policy is better than the baseline, which should be awarded to use more. On the contrary, if ${\widehat{A}}_{t}<0$ , it says that the current policy is worse, and it should have a punishment. However, the adjust of policy ratio should within (1-$\varepsilon$,1 + $\varepsilon$) to maintain the two policies not differ much.

### Two biological motions in nature

**Figure 1:**
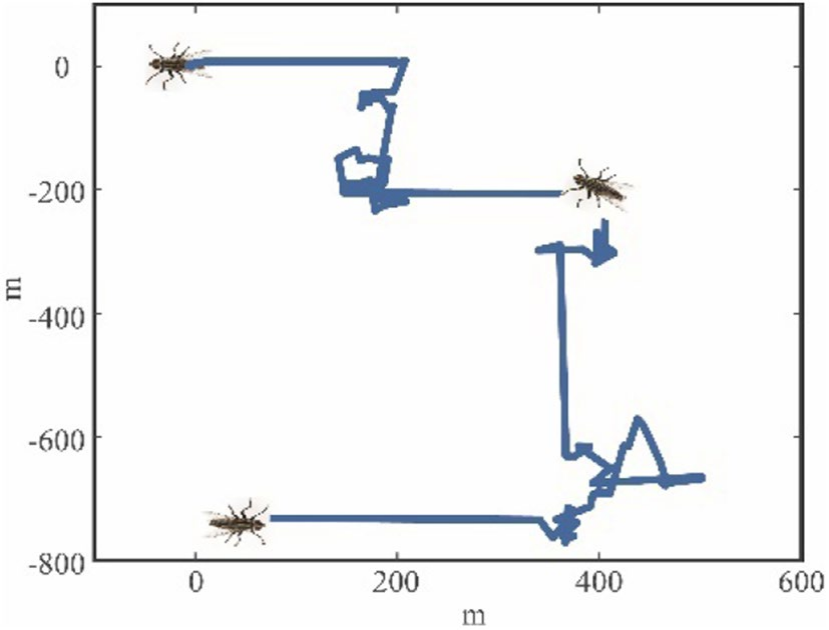
The Levy movement of a drosophila melanogaster.

In nature, creatures often have their own movement strategies to prey on or avoid enemies. Here, two kinds of motion laws are mainly concerned about: Levy motion and Brown motion. Levy motion is a mode of random walking, the livings with Levy motion generally have unpredictable traces. One of the typical representatives of Levy motion is the fly. The movement of a fly in the air is usually unpredictable, making it usually difficult to catch and strike by predicting its movement. This result is due to the characteristics of Levy motion. Taking one kinds of flies, drosophila melanogaster, as the example, the movement is simulated in Fig. 1.

**Figure 2:**
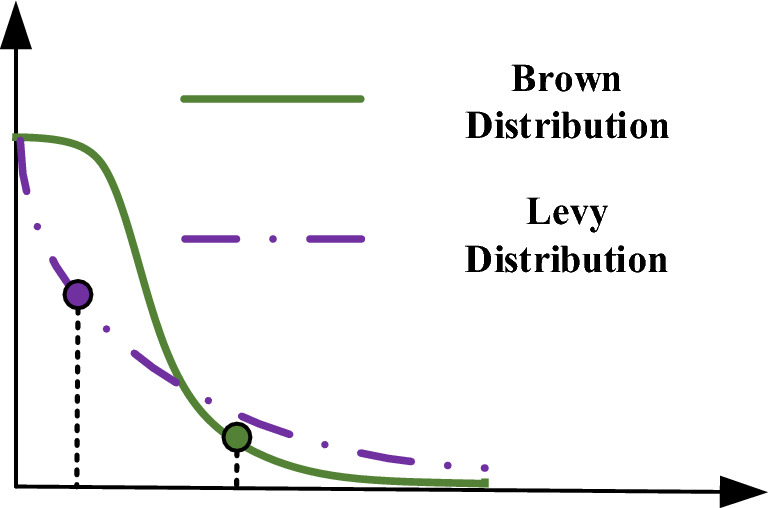
The corresponding distribution forms of Levy motion and Brown motion.

It can be seen from Fig. 1 that the fly using Levy motion will maintain straight flights and large angles turning at the same time when moving. It is for this reason that the trajectory of the fly is elusive. It is found that there exists a mathematical law behind this phenomenon, namely, Levy motion, which is a power-law distribution shown in Fig. 2. Due to the power-law distribution, most of the moving steps of the livings will be very short, but some will be obviously long.

In addition to Levy motion, another kind of random walking that we concentrate on is namely as Brown motion. In a Brown motion, the movement steps are concentrated in a region, which basically conforms to a Gaussian distribution, and Fig. 2 also shows this distribution of Brown motion.

**Figure 3:**
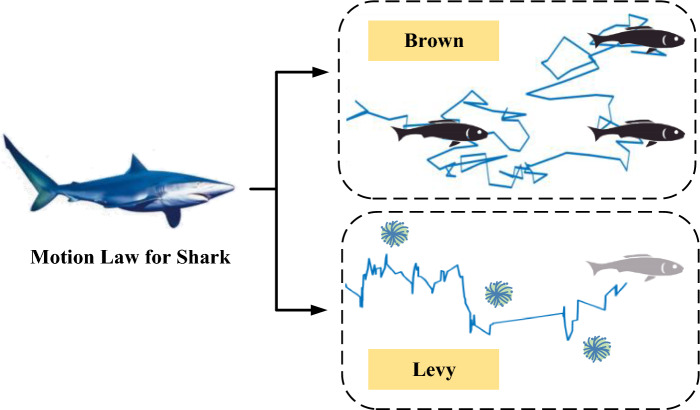
The motion law for the shark based on the hybrid of Brown and Levy motions.

According to the analysis of the laws in Fig. 2, Levy motion is more effective than Brown motion in exploration under the same condition. In fact, it is true that Levy motion is generally adopted to explore for the creatures living in those scarce resources of environments. It is worth noting that, in some cases, the two motions can be combined together as a hybrid one. Taking a typical marine predator, shark, for example, it takes the combination of two motions, shown in Fig. 3.

It can be seen that the movement for the shark can be divided into two types. For the first type, when it is known that there is food nearby, the shark will take a Brown motion. With the advantage of Brown motion, the scattered and hidden food can be found in a small area around the shark, which is a suitable way for shark to clean up the food nearby. However, when there is a shortage of food, a new territory needs to be developed, and the second type is needed. In the second type, the shark turns to Levy motion. Due to the nature of Levy motion, the movement coverage area will increase, which will help the shark to expand the movement range and find the potential food.

### Basis of quantum computation

Quantum genetic algorithm (QGA) is a probabilistic evolutionary algorithm with quantum characteristics formed by the cross fusion of quantum computing and genetic algorithm^39,40^. It uses a group of independent quantum bits with superposition property to encode, update and evolve through the quantum rotation gate, thus realizing the rapid solution of the optimization problem. QGA itself has the advantages of genetic algorithm; at the same time, it can make up for the lack of population diversity because of the quantum superposition property. Thus, compared with conventional GA, it has a better optimization effect. The basic unit of storage in quantum computing is the quantum bit (qubit). The qubit can make the quantity in 0 state, 1 state or a superposition state, and such the property is called as quantum superposition.

A qubit can be represented by Dirac notation as follows

5 $$\left|\psi \right.\rangle =\alpha \left|0\right.\rangle +\beta \left|1\right.\rangle $$

where $\alpha$ and $\beta$ are the probability amplitudes for $\left|0\right.\rangle$ and $\left|1\right.\rangle$, respectively. A normalization condition needs to be met here.

6 $${\left|\alpha \right|}^{2}+{\left|\beta \right|}^{2}=1$$

The quantum superposition state is not a definite state, but a linear superposition of the ground states. When the outside world conducts an observation, it will collapse to a certain state with a probability amplitude.

For a single qubit, there are mainly two ground states: $\left|0\right.\rangle$ and $\left|1\right.\rangle$. If it is a multiple quantum bit, it is essentially composed of multiple single qubits. In the case of a double quantum bit, it will contain four ground states, including $\left|00\right.\rangle$, $\left|01\right.\rangle$, $\left|10\right.\rangle$, and $\left|11\right.\rangle$. The quantum state of a double quantum bit can be expressed as

7 $$\left|\psi \right.\rangle ={a}_{00}\left|00\right.\rangle +{a}_{01}\left|01\right.\rangle +{a}_{10}\left|10\right.\rangle +{a}_{11}\left|11\right.\rangle $$

where ${a}_{00}$, ${a}_{01}$, ${a}_{10}$ and ${a}_{11}$ represent the probability amplitudes. In details, ${\left|{a}_{00}\right|}^{2}$, ${\left|{a}_{01}\right|}^{2}$, ${\left|{a}_{10}\right|}^{2}$ and ${\left|{a}_{11}\right|}^{2}$ are the probabilities of collapsing to $\left|00\right.\rangle$, $\left|01\right.\rangle$, $\left|10\right.\rangle$ and $\left|11\right.\rangle$, respectively. Certainly, they also need to meet the normalization conditions.

8 $${\left|{a}_{00}\right|}^{2}+{\left|{a}_{01}\right|}^{2}+{\left|{a}_{10}\right|}^{2}+{\left|{a}_{11}\right|}^{2}=1$$

Since a double quantum bit is composed of two single qubits, the probability of observing the 0 state for the first single qubit is ${\left|{a}_{00}\right|}^{2}+{\left|{a}_{01}\right|}^{2}$, and the state after the observing is as below.

9 $$\left|{{\psi }_{1}}^{\mathrm{^{\prime}}}\right.\rangle =\frac{{a}_{00}\left|00\right.\rangle +{a}_{01}\left|01\right.\rangle }{\sqrt{{\left|{a}_{00}\right|}^{2}+{\left|{a}_{01}\right|}^{2}}}$$

In the same way, the probability that the first single qubit is observed as a state of 1 is ${\left|{a}_{10}\right|}^{2}+{\left|{a}_{11}\right|}^{2}$, and the observed state is expressed as below.

10 $$\left|{{\psi }_{1}}^{\mathrm{^{\prime}}}\right.\rangle =\frac{{a}_{10}\left|10\right.\rangle +{a}_{11}\left|11\right.\rangle }{\sqrt{{\left|{a}_{10}\right|}^{2}+{\left|{a}_{11}\right|}^{2}}}$$

Besides, the probabilities that the second single qubit is observed as a state of 0 and 1 are ${\left|{a}_{00}\right|}^{2}+{\left|{a}_{10}\right|}^{2}$ and ${\left|{a}_{01}\right|}^{2}+{\left|{a}_{11}\right|}^{2}$, with the observed states shown as below.

11 $$\left|{{\psi }_{2}}^{\mathrm{^{\prime}}}\right.\rangle =\frac{{a}_{00}\left|00\right.\rangle +{a}_{10}\left|10\right.\rangle }{\sqrt{{\left|{a}_{00}\right|}^{2}+{\left|{a}_{10}\right|}^{2}}}$$

12 $$\left|{{\psi }_{2}}^{\mathrm{^{\prime}}}\right.\rangle =\frac{{a}_{01}\left|01\right.\rangle +{a}_{11}\left|11\right.\rangle }{\sqrt{{\left|{a}_{01}\right|}^{2}+{\left|{a}_{11}\right|}^{2}}}$$

Broadly speaking, for an *n*-qubit system, there will be ${2}^{n}$ ground states, and the probability amplitude of each ground state should also meet the normalization condition in Eq. (8). To complete a quantum computation in QGA, quantum logic gates are also required to process information. The gates used in this paper are mainly quantum rotation ones, which will be introduced in Section “External Parameter optimization based on quantum genetic algorithm”.

## Overall design of the multiple-interpretable improved PPO algorithm with few-shot technique

Facing to a planar tracking problem, the multiple-interpretable improved PPO algorithm with few-shot technique proposed in this paper mainly includes two aspects. On the one hand, it contains an improvement in interpretability. Specifically, it will integrate the interpretabilities into the algorithm from three levels. On the other hand, it contains an attempt to find a fast generation way of fake samples to reduce the demand of real interactive samples. The following will discuss these two points separately.

### Design of GBQ-PPO

**Figure 4:**
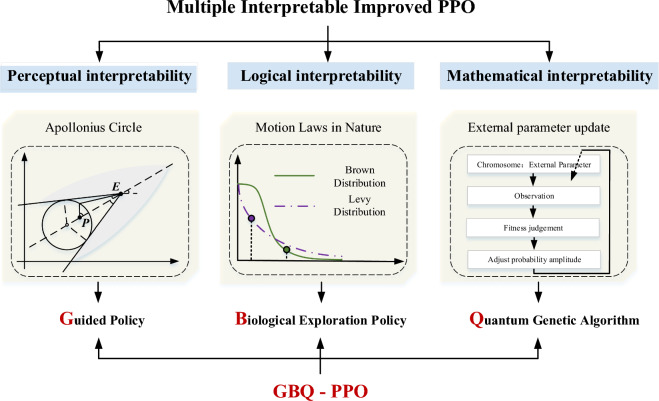
The diagram of the proposed multiple-interpretable improved PPO.

Compared with the typical PPO, the multiple-interpretable improved PPO algorithm is characterized by different improvements on multiple levels of interpretability. Since the concept of interpretability can be divided into three levels of perception, logic and mathematics, the proposed algorithm will also be improved at these three levels. Figure 4 shows the framework of the algorithm. Due to the reason that the improvements are mainly brought about the introductions of guided policy, biological exploration policy and employment of the quantum genetic algorithm, this algorithm is denoted as GBQ-PPO later in this paper.

*Firstly, for the part of perceptual interpretability, a guided policy based on Apollonius circle is added*. Compared with the method that directly optimizing the parameters in RL, the guided policy will help the agent to obtain useful samples at the very early stage of learning and avoid the invalid explorations. In addition, because the Apollonius circle is strictly algebraic derived, it has a good interpretability, as people can easily verify its correctness. In other words, the introduction of the guided policy will help people recognize the rationality of the algorithm composition from a perceptual level, thus enhancing the perceptual interpretability. *Secondly, for the logical interpretability part, the biological Brown and Levy motions are introduced to form a new hybrid exploration policy*, so as to reconstruct forms of the new and old policies in PPO. The corresponding interpretability of the algorithm is given by the biological characteristics. Therefore, the algorithm is interpretable at the overall logical level of agent exploration and update. *Thirdly, for the mathematical interpretability, the concept of external parameter update is introduced except for the internal parameter update*. The existence of external parameters appears from the aforementioned introductions of guided policy and biological exploration policy. The selection of different parameters may affect the performance; therefore, coding these external parameters and optimizing them based on the QGA method will form a new loop of external parameter update, which is independent from the internal policy parameter update. Since QGA itself is an interpretable evolutionary algorithm, the update of external parameters will also give the algorithm interpretability at a mathematical level.

### F-GBQ-PPO: Combination of few-shot technique

**Figure 5:**
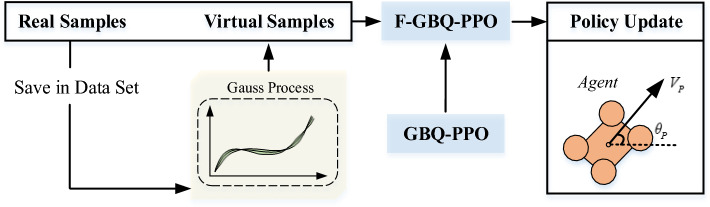
The diagram of the few-shot technique involved in F-GBQ-PPO.

Except for improving the interpretabilities, this paper also proposes a method that can quickly generate fake samples. Different from GAN and meta-learning methods, which need to simulate the distribution of real samples or find necessary meta parameters, our method can generate fake samples more quickly, and it is based on a multi-dimensional Gaussian process. After mixing the fake samples and the real ones, the agent can normally conduct the policy update, shown in Fig. 5.

In Fig. 5, it is seen that the agent can obtain real samples in the process of interaction with the environment. By storing these samples, a dataset can be generated. Based on this dataset, the future states can be predicted based on a multi-dimensional Gaussian process, thus a series of fake samples can be generated. In this way, the consumption of real samples can be saved. Since the multiple-interpretable improved PPO is defined as GBQ-PPO, the version that introduces the few-shot technique can be recorded as F-GBQ-PPO. Thanks to the powerful fitting ability of the multi-dimensional Gauss distribution, the future states can be quickly calculated, and the specific process will be described in Section "Few-shot technique based on Gaussian process prediction". In this way, the generation of fake samples will be quite quick and convenient, because it does not need a training process. Certainly, the method is not without disadvantages compared with other few-shot ways. In fact, we only try to reduce the demand for real samples to a certain extent, but cannot achieve zero samples (refers to the situation where an agent does not need to continue to provide them after a certain number of real samples are fed). Due to the characteristics of multi-dimensional Gauss distribution, real samples are always need to generate fake samples, so as to maintain that the fake samples do not deviate too much from the real ones. Thus, our proposed F-GBQ-PPO updates the dataset timely during the training to keep the effectiveness of the few-shot part.

## Components of the proposed method

For the proposed algorithm of multiple-interpretable improved PPO with few-shot technique, the specific components can be divided into four parts. Among these parts, the first three ones are about the improvements of three interpretable levels, and the fourth part is about the description of few-shot technique. The following contents will introduce the four parts in turn.

### Guided policy based on the Apollonius circle

**Figure 6:**
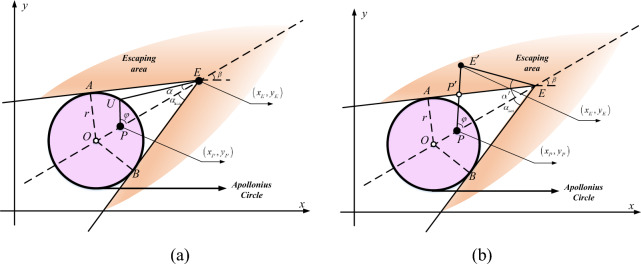
The geometric illustration of Apollonius circle.

For a planar tracking problem, if the speeds of the participants are constant and can be known, the tracking strategy can usually be found using an Apollonius circle. An Apollonius circle will divide the space into a tracking area and an untraceable area (or escaping area) according to the actual position and speed of participants. For the pursuer, the goal is always to stay within the tracking area; besides, for that who wants to escape, it should try best to stay in the escaping area. Suppose a one-to-one scenario, and denote the pursuer as *P* and the evader as $E$. Set the positions of $P$ and $E$ are $({x}_{P}, {y}_{P})$ and $({x}_{E}, {y}_{E})$, respectively. The Apollonius circle is the one that centered at $O$ with the radius $r$, which is shown in Fig. 6.

In this scenario, the pursuer $P$ and the evader $E$ hold the constant speeds ${V}_{p}$ and ${V}_{e}$, respectively. Therefore, the factor $\zeta$ is defined to represent the speed ratio, shown as

13 $$ \zeta = \frac{PU}{{EU}} = \frac{{V_{p} }}{{V_{e} }} $$

where $\zeta \in \left(\mathrm{0,1}\right)$. In the figure, there exist a $U$ point, which is located on the circle, and all the potential $U$ points will form the whole scale of the Apollonius circle. It means that if $E$ moves to a $U$ point, $P$ can capture it by moving to $U$. Based on the geometric analysis, the expressions of the center $O$ and the radius $r$ can be calculated, and shown in Eqs. (14) and (15).

14 $$ O = \frac{{x_{P} - \zeta^{2} x_{e} }}{{1 - \zeta^{2} }},\frac{{y_{P} - \zeta^{2} y_{e} }}{{1 - \zeta^{2} }} $$

15 $$r= \frac{\zeta \sqrt{{({x}_{P}-{x}_{e})}^{2}+ {({y}_{P}-{y}_{e})}^{2} }}{1 - {\zeta }^{2}}$$

It is noticed that in an Apollonius circle, if the speed ratio $\zeta$ is getting smaller, the circle radius $r$ will be decreased and as a result, the region of Apollonius circle will also get smaller. In this condition, the evader will have a larger escaping area. It is seen that when the evader moves to a $U$ point, it will be captured; therefore, the area where the direction can meet this condition can be defined as a tracking area. Obviously, the area has a sector angle. In Fig. 6, this angle is *∠AEB*, which consists two of ${\alpha }_{max}$. The symbol $\alpha$ represents the angle between the line-of-sight and the evader’s heading direction, and its max value can be obtained as below.

16 $${\alpha }_{max}={\text{arcsin}}\left(\frac{{V}_{p}}{{V}_{e}}\right)=\mathrm{ arcsin}\left(\zeta \right)$$

As shown in Fig. 6a, when the angle $\alpha$ is not greater than ${\alpha }_{max}$ , the pursuer $P$ can always find an angle $\varphi$ that ensures the capture of $E$. Thus, the sector angle for the capturing area can be obtained, denoted as $\eta$.

17 $$\eta = {2\alpha }_{max}=2\mathit{arcsin}\left(\zeta \right)$$

Therefore, based on the Apollonius circle, it is concluded that if the moving angle of $E$ is $\beta -\alpha +\pi$ ($\alpha <{\alpha }_{max})$ , the responded moving angle of $P$ will be $\beta + \varphi$ along with $\mathop{PU}\limits^{\rightharpoonup}$. The optimal moving strategy for $P$ can be obtained as below.

18 $${\theta }_{P}= \beta +\varphi = {{\text{sin}}}^{-1}(\frac{{\text{sin}}\alpha }{\zeta })+\beta (\alpha <{\alpha }_{max})$$

When the condition comes to ${\alpha }_{max}< \alpha < \pi$, the pursuer $P$ cannot capture the evader $E$. However, if $P$ still moves with the directional angle $\beta + \varphi$, it will get as close as possible to $E$, which can be seen from Fig. 6b. In the figure, it is seen that if $E$ moves to $E\mathrm{^{\prime}}$, the optimal movement for $P$ will be heading to $E\mathrm{^{\prime}}$. Therefore, if ${\alpha }_{max}< \alpha < \pi$, the optimal moving strategy for $P$ can be obtained by

19 $${\theta }_{P}= \beta + \varphi = {\mathit{cot}}^{-1}\left[\frac{\left|EP\right|}{{V}_{e}\Delta t\mathit{sin}\alpha }- \mathit{cot}\alpha \right]+\beta $$

where $\Delta t$ is the time interval.

To sum up, the optimal strategy for the pursuer $P$ should be as follows.

20 $$ \theta _{P}  = \beta  + \varphi  = \left\{ {\begin{array}{*{20}l}    {\beta  + \sin ^{{ - 1}} (\frac{{\sin \alpha }}{\zeta })} \hfill & {if~~~\alpha  \le \alpha _{{max}} } \hfill  \\    {\beta  + \cot ^{{ - 1}} \left[ {\frac{{\left| {E_{0} P_{i} } \right|}}{{V_{e} {{\Delta }}t\sin \alpha }} - \cot \alpha } \right]} \hfill & {if~~~~\alpha _{{max}}  < ~\alpha  < ~\pi } \hfill  \\   \end{array} } \right. $$

It is noticed that the Apollonius circle is obtained based on the correct information of the participants. That says, if the information is not accurate enough, the optimal policy ${\theta }_{P}$ will be not effective enough.

Although it is hard to get the accurate information of an environment, one can get an estimated model according to the human knowledge of planar tracking. In this way, the policy ${\theta }_{P}$ which is generated from the estimated model, can be employed as a guided policy for the agent in real environment to get beneficial samples and avoid meaningless explorations at the early stage of learning. Suppose that the action generated from RL policy is $a$, thus the actual output of the agent is

21 $$a\mathrm{^{\prime}} = (1-m)\times a + {m\times \theta }_{P}$$

where $m$ represents the ratio parameter of the guided policy, determining how much that we can believe in the policy generated from Apollonius circle.

Certainly, it would be hard to select an appropriate value of $m$, because it is hard to know the difference between the real environment and our estimated one. Therefore, $m$ will be set as an external parameter, as a part of the external loop in the algorithm.

### Hybrid interpretable exploration policy in PPO

In PPO algorithm, there usually exist two different policies $\pi$ and $\pi {\prime}$, where $\pi$ is used to interact with the environment and $\pi {\prime}$ is the one that needs to be updated. Generally, the two policies obey Gauss distributions for a continuous problem.

However, in the past, the distribution for policy was set to obey the Gaussian distribution only out of habit, but the reason for this was not analyzed in depth. Here, Levy and Brown motions in nature will be introduced to redesign the distribution forms for the exploration policy.

Since Brown motion obeys the form of Gauss distribution ${\mathbb{N}}(\phi {(s)}^{\text{T}}\theta ,{\sigma }^{2})$, the policy $\pi {\prime}$ that need to be updated can be seen as a type of Brown motion, and can be expressed as

22 $${\pi {\prime}}_{\theta }(a|s)=\frac{1}{\sqrt{2\pi }\sigma }{\text{exp}}(-\frac{(a-\phi {(s)}^{\text{T}}\theta )}{2{\sigma }^{2}})$$

and the generated action *a* is

23 $$a={\mu }_{\theta }+\varepsilon =\phi {(s)}^{\text{T}}\theta +\varepsilon $$

where $\theta$ is the corresponding parameters for RL policy. As for the exploration policy $\pi$ , we are inspired by the predatory strategy of the shark, and will integrate Brown motion and Levy motion to form a new hybrid exploration policy. Based on the description in Section “Two biological motions in nature”, it is known that Levy motion conforms to a power-law distribution, which is different from Brown motion. For correctly expressing the characteristics for Levy motion, the Pareto distribution is applied.

If $X$ is a random variable with a Pareto distribution, the cumulative distribution function of a Pareto random variable with parameter $\alpha$ and ${x}_{m}$ is as follows.

24 $$ F_{{\left( {x_{m} ,\alpha } \right)}} \left( x \right) = \left\{ {\begin{array}{*{20}l}    {1 - \left( {x_{m} /x} \right)^{\alpha } ,} \hfill & {x \ge x_{m} } \hfill  \\    {0,} \hfill & {x < x_{m} } \hfill  \\   \end{array} } \right. $$

This Pareto distribution is characterized by a scale parameter ${x}_{m}$ and a shape parameter $\alpha$, which is known as the tail index. Besides, the probability density function is as follows.

25 $$ f_{{\left( {x_{m} ,\alpha } \right)}} \left( x \right) = \left\{ {\begin{array}{*{20}l}    {x_{m}^{\alpha } /x^{{\alpha  + 1}} ,} \hfill & {x \ge x_{m} } \hfill  \\    {0,} \hfill & {x < x_{m} } \hfill  \\   \end{array} } \right. $$

Since Brown motion and Levy motion are from nature, the hybrid exploration policy based on the two motions will be expected to have the interpretability from the view of biology. Therefore, policy $\pi$ that the agent adopts to interact with the environment is redesigned as the hybrid exploration policy, expressed as

26 $$\pi =q\cdot {\pi }_{L}+(1-q)\cdot {\pi }_{B}$$

where ${\pi }_{L}$ and ${\pi }_{B}$ represent the policy that generates from Pareto distribution and Gauss distribution, respectively; and $q$ represents the hybrid parameter. It is seen that different values of $q$ will make the policy have different performances. Because Brown motion is more suitable for small-scale exploration while Levy motion works better when the exploration needs a wide range, the agent can switch between the two motions according to the task it meets by selecting an appropriate parameter $q$.

In this way, the forms of policies in PPO are redesigned, and the new hybrid exploration policy has the interpretability from the biological view, making the algorithm logical interpretable. Conventionally, in a PPO algorithm, it is necessary to maintain the two policies not differ too much. However, it is confused that what is the criterion for the difference. In the past research, people give a limit (like *KL* divergence or $\varepsilon$ in PPO2) to achieve this goal. However, it is still confused that how to determine a limit. In our proposed GBQ-PPO, the hybrid parameter $q$ will be set as one of external parameters, together with the parameter $m$, building up an external loop for the agent.

### External Parameter optimization based on quantum genetic algorithm

The external parameters $m$ and $q$ are introduced due to the employments of guided policy and hybrid exploration policy. Further, what we need to do is to find the optimal values of the two parameters which will bring the most reward for the agent. Here, the quantum genetic algorithm (QGA) is applied. Compared with normal genetic algorithm (GA), QGA uses the coding of qubits to make a chromosome. As a result, QGA can express the superposition of multiple states at the same time, making the effect better than the normal GA.

Similarly like a typical GA process, the chromosome coding is generally required. In QGA, this encoding is realized by qubit pairs. A system with a *n*-qubit system can be expressed as

27 $$\left|\begin{array}{c}{\alpha }_{1}\\ {\beta }_{1}\end{array}\right|\left|\begin{array}{c}{\alpha }_{2}\\ {\beta }_{2}\end{array}\right|\begin{array}{c}\cdots \\ \cdots \end{array}\left|\begin{array}{c}{\alpha }_{n}\\ {\beta }_{n}\end{array}\right|$$

where ${\alpha }_{i}$ and ${\beta }_{i}$ need to meet the normalization conditions.

28 $${|{\alpha }_{i}|}^{2}+{|{\beta }_{i}|}^{2}=1(i=\mathrm{1,2},\cdots ,n)$$

The system can be represented by the linear superposition of ${2}^{n}$ ground states, representing all states in the form of probability. After one time of observation, the state will collapse into a certain one with probability, and the probability of collapsing depends on the probability amplitudes in the state.

Except for the application of above quantum computation, the quantum rotation gate is also need in QGA to change the probability amplitude. Through the interaction of each quantum superposition state, the chromosome will be updated so as to optimize the external parameters. The definition of a quantum rotation gate is

29 $$R\left(\theta \right)=\left[\begin{array}{cc}\mathit{cos}\theta & -\mathit{sin}\theta \\ \mathit{sin}\theta & \mathit{cos}\theta \end{array}\right]\left[\begin{array}{c}\alpha \\ \beta \end{array}\right]=\left[\begin{array}{c}{\alpha }^{\mathrm{^{\prime}}}\\ {\beta }^{\mathrm{^{\prime}}}\end{array}\right]$$

A quantum superposition state $\left|\psi \right.\rangle =\alpha \left|0\right.\rangle +\beta \left|1\right.\rangle$ can be obtained by rotating the gate

30 $$\left|{\psi }^{\mathrm{^{\prime}}}\right.\rangle =R\left(\theta \right)\times \left|\psi \right.\rangle =\left[\begin{array}{cc}\mathit{cos}\theta & -\mathit{sin}\theta \\ \mathit{sin}\theta & \mathit{cos}\theta \end{array}\right]\left[\begin{array}{c}\alpha \\ \beta \end{array}\right]=\left[\begin{array}{c}{\alpha }^{\mathrm{^{\prime}}}\\ {\beta }^{\mathrm{^{\prime}}}\end{array}\right]$$

where $\theta$ represents the rotation angle. In Eq. (30), $$\left[\begin{array}{c}\alpha \\ \beta \end{array}\right]$$ is the probability amplitude of quantum superposition state before the rotation, and $$\left[\begin{array}{c}{\alpha }^{\mathrm{^{\prime}}}\\ {\beta }^{\mathrm{^{\prime}}}\end{array}\right]$$ is the one that after the update by the gate. Obviously, the quantum superposition state $\left|\psi \right.\rangle$ rotates $\theta$ through the action of rotation gate to obtain $\left|{\psi }^{\mathrm{^{\prime}}}\right.\rangle$.

Define the population as $Q\left(t\right)=\left\{{q}_{1}^{t},{q}_{2}^{t},\cdots ,{q}_{m}^{t}\right\}$, where $m$ represents the population size, and $t$ represents the current evolutionary generation. For an individual ${q}_{j}^{t}$, it can be defined as a kind of *n*-qubit system $${q}_{j}^{t}=\left|\begin{array}{c}{\alpha }_{1}^{t}\\ {\beta }_{1}^{t}\end{array}\right|\left|\begin{array}{c}{\alpha }_{2}^{t}\\ {\beta }_{2}^{t}\end{array}\right|\begin{array}{c}\cdots \\ \cdots \end{array}\left|\begin{array}{c}{\alpha }_{n}^{t}\\ {\beta }_{n}^{t}\end{array}\right|$$. By initializing all probability amplitudes ${\alpha }_{i}^{t}$ and ${\beta }_{i}^{t}$ equal to $\frac{1}{\sqrt{2}}$ , it makes sure that the observation can collapse to a certain state with equal probability under initialization. The $t$ th-generation solution ${x}_{j}^{t}\left(j=\mathrm{1,2},\cdots ,m\right)$ can be obtained by observing the population $Q$. Define ${x}_{j}^{t}\left(i\right)$ as the $i$ th position on chromosome for ${q}_{j}^{t}$, $b\left(i\right)$ as the $i$ th position on the current best chromosome, and $f\left(x\right)$ as the fitness function. Through comparing the values of $f\left(x\left(i\right)\right)$ and $f\left(b\left(i\right)\right)$, the probability amplitudes ${\left[{\alpha }_{i},{\beta }_{i}\right]}^{T}$ can be adjusted by making individuals evolve in a favorable direction. Define $s\left({\alpha }_{i},{\beta }_{i}\right)$ as the rotation direction and $\Delta {\theta }_{i}$ as the angle value of rotation, thus the specific rotation angle with direction ${\theta }_{i}=s\left({\alpha }_{i},{\beta }_{i}\right)\Delta {\theta }_{i}$ can be obtained. In this way, the population is continuously updated and the final best chromosome is found.

For the external parameters $m$ and $q$, a two-bit qubit system is need to find the optimized values. Through Eqs. (27)–(30) and the above update process, the optimal $m*$ and $q*$ can be obtained. In this way, the update of policy not only contains the parameter in the policy network, but also contains the parameters $m$ and $q$ in the external loop. In this way, there are more possibilities for the agent to choose its actions, thus it has the ability to greatly improve the learning efficiency and expand the exploration space.

### Few-shot technique based on Gaussian process prediction

**Figure 7:**
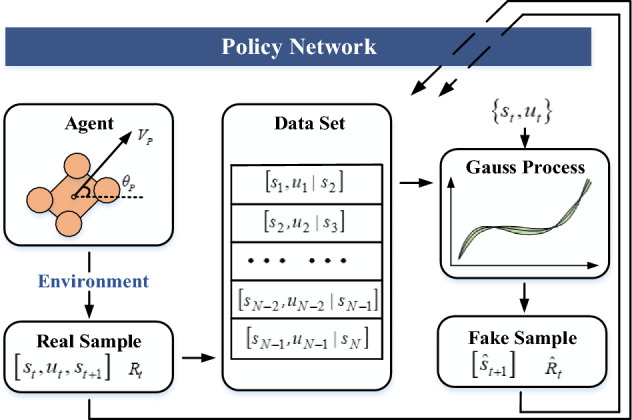
The generation process of fake samples based on Gaussian process.

Except for the improvements on interpretabilities, another innovation of this paper is to reduce the demand for real samples by generating fake samples. In order to achieve this goal, the multi-dimension Gaussian process (GP) is used for prediction. To conduct the prediction, it is necessary to build a GP dataset. The dataset is composed of different tuples of state-action pairs, which can be arrayed from real samples, shown in Fig. 7.

Suppose that the state ${s}_{t}$ satisfies the following equation

31 $${s}_{t}=f\left({s}_{t-1},{u}_{t-1}\right)$$

which means that the state ${s}_{t}$ can be determined based on previous state ${s}_{t-1}$ and action ${u}_{t-1}$. The main problem is that the relationship function $f\left(\cdot \right)$ is unknown. The main concept of Gaussian process is to predict state ${s}_{t}$ based on the previous state-action pairs.

To conduct a prediction process, the state-action pairs and the corresponding training targets are required. Denote ${\widetilde{w}}_{t-1}=\left({s}_{t-1},{u}_{t-1}\right)$ as the state-action pair at $t-1$, and ${\Delta }_{t}={s}_{t}-{s}_{t-1}+\varepsilon$ as the training target. Therefore, the dataset is designed as

32 $$D:=\left\{{{\widetilde{W}}_{t-1}:=[{\widetilde{w}}_{1},...,{\widetilde{w}}_{n}]}^{T}, Y:={[{\Delta }_{1},...,{\Delta }_{n}]}^{T}\right\}$$

where $\varepsilon \sim N\left(0,{\Sigma }_{\varepsilon }\right), { \Sigma }_{\varepsilon }=diag\left(\left[{\sigma }_{\varepsilon 1},...,{\sigma }_{\varepsilon D}\right]\right)$. In this way, the GP dataset is maintained. Further, based on this dataset, it is possible to obtain the state distribution of ${s}_{t}$

33 $$P({s}_{t}|{s}_{t-1},{u}_{t-1})= N\left({s}_{t}|{\mu }_{t},{\Sigma }_{t}\right)$$

where ${\mu }_{t}$ and ${\Sigma }_{t}$ represent the mean and variance of the distribution respectively, shown as below.

34 $${\mu }_{t}={s}_{t-1}+{E}_{f}\left[{\Delta }_{t}\right] {\Sigma }_{t}=var\left[{\Delta }_{t}\right]$$

Therefore, the problem can be changed to obtain ${E}_{f}\left[{\Delta }_{t}\right]$ and $var\left[{\Delta }_{t}\right]$. For this goal, the kernel function for $f\left(\widetilde{w}\right)$ is necessary, which can be designed as

35 $$\mathit{cov}\left(f\left(\widetilde{w}\right),f\left(\widetilde{w}\mathrm{^{\prime}}\right)\right)={\alpha }^{2}\mathit{exp}\left(-\frac{{\Vert \widetilde{w}-\widetilde{w}\mathrm{^{\prime}}\Vert }^{2}}{2{l}^{2}}\right)=ker\left(\widetilde{w},\widetilde{w}\mathrm{^{\prime}}\right)$$

where $\alpha$ and $l$ are the scale factors. The specific values for $\alpha$ and $l$ can be optimized through maximizing a marginal log-likelihood function, expressed as

36 $$\mathit{log}P\left(Y\left|\alpha ,l\right.\right)=-\frac{1}{2}{Y}^{T}{{K}_{yy}}^{-1}Y-\frac{1}{2}\mathit{log}\left|{K}_{yy}\right|-\frac{n}{2}\mathit{log}\left(2\pi \right)$$

where ${K}_{yy}$ is the covariance matrix generated from $ker\left(\widetilde{w},\widetilde{w}{\prime}\right)$. Further, the kernel function for $\Delta \left(\widetilde{w}\right)$ is attained as below.

37 $$ \begin{aligned} cov\left( {\Delta \left( {\tilde{w}} \right),\Delta \left( {\tilde{w}^{\prime}} \right)} \right) = & cov\left( {f\left( {\tilde{w}} \right) + \varepsilon ,f\left( {\tilde{w}^{\prime}} \right) + \varepsilon } \right) \\ = & cov\left( {f\left( {\tilde{w}} \right),f\left( {\tilde{w}^{\prime}} \right)} \right) + {\text{cov}} \left( {\varepsilon ,\varepsilon } \right) \\ = & ker\left( {\tilde{w},\tilde{w}^{\prime}} \right) + \sigma_{\varepsilon }^{2} I \\ \end{aligned} $$

Suppose that the information ${\widetilde{W}}_{t-1}$, $Y$ are known and obey multi-dimension Gaussian distributions. As a result, if the input is $w*$ , the joint probability distribution should also be Gaussian, shown in Eq. (38).

38 $$\left[\begin{array}{c}Y\\ \Delta \left(w*\right)\end{array}\right]\sim N\left(\left[\begin{array}{c}0\\ 0\end{array}\right],\left[\begin{array}{cc}K+{\sigma }_{\varepsilon }^{2}I& {k}_{*}\\ {k}_{*}^{T}& {k}_{**}\end{array}\right]\right)$$

It is noticed that there is no experience in $Y$ and $w*$, their output mean values are zero. Besides, in Eq. (28), the following relations exist.

39 $${k}_{*}=ker\left(\widetilde{W},w*\right) {k}_{**}=ker\left(w*,w*\right) {K}_{ij}= ker\left({\widetilde{w}}_{i},{\widetilde{w}}_{j}\right).$$

Based on Bayesian posterior formula, it is known that

40 $$P\left(Y,\Delta \left(w*\right)\right)=P\left(\Delta \left(w*\right)|Y\right)P\left(Y\right)$$

where $P\left(Y,\Delta \left(w*\right)\right)$ is the distribution that we focus on. After the necessary deductions, this distribution can be found that obeys

41 $$P\left(\Delta \left(w*\right)|Y\right)\sim N\left({k}_{*}^{T}{\left(K+{\sigma }_{\varepsilon }^{2}I\right)}^{-1}y,{k}_{**}-{k}_{*}^{T}{\left(K+{\sigma }_{\varepsilon }^{2}I\right)}^{-1}{k}_{*}\right)$$

with the mean ${m}_{f}\left(w*\right)$ and the variance ${\sigma }_{f}^{2}\left(\Delta \left(w*\right)\right)$ expressed as follows.

42 $${m}_{f}\left(w*\right)={E}_{f}\left[\Delta \right]={k}_{*}^{T}{\left(K+{\sigma }_{\varepsilon }^{2}I\right)}^{-1}y={k}_{*}^{T}\beta $$

43 $${\sigma }_{f}^{2}\left(\Delta \left(w*\right)\right)=\mathit{var}\left[\Delta \right]={k}_{**}-{k}_{*}^{T}{\left(K+{\sigma }_{\varepsilon }^{2}I\right)}^{-1}{k}_{*}$$

Therefore, based on the results in Eqs. (42) and (43), the distribution $P({s}_{t}|{s}_{t-1},{u}_{t-1})$ shown in Eq. (33) can be obtained.

Certainly, the predicted value of ${s}_{t}$ is not the real state that the agent experienced. Therefore, the symbol ${\widehat{s}}_{t}$ is defined to represent the fake state differing from the real state ${s}_{t}$. According to the reward function, the predicted reward ${\widehat{R}}_{t}$ can be calculated, where a tuple $\langle {\widehat{s}}_{t},{\widehat{R}}_{t}|{s}_{t-1},{u}_{t-1}\rangle$ can be generated as a fake sample. The whole diagram for this few-shot technique is shown in Fig. 7.

From the figure, it is seen that when the agent interacts with the environment, the real sample $\left[{s}_{t},{u}_{t}\right]$ can be available. Through saving these real samples in a stack, the dataset for GP is built. Through a multi-dimension GP process, the mean ${E}_{f}\left[{\Delta }_{t}\right]$ and variance $\mathit{var}\left[{\Delta }_{t}\right]$ of the output can be predicted, and then the predicted state ${\widehat{s}}_{t+1}$ and reward ${\widehat{R}}_{t}$ are obtained. Based on the real samples and the fake samples, the agent can get the policy updated while reducing the demand for real interactive samples.

To sum up, the overall flow of the proposed method is expressed as follows.

**Algorithm 1:**
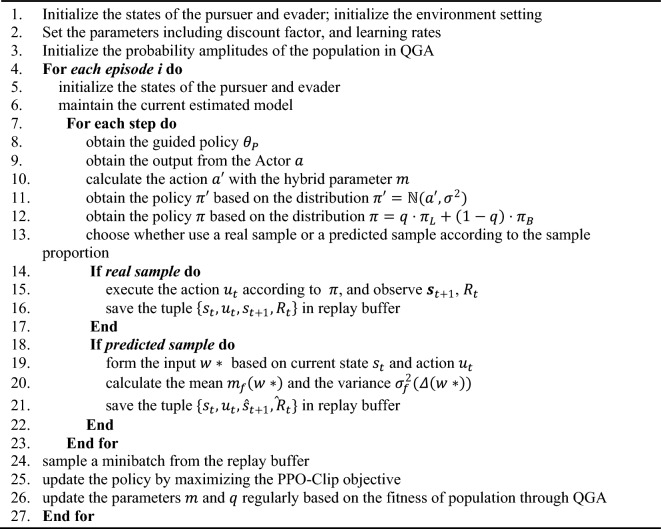
The multiple-interpretable improved PPO algorithm with few-shot technique

## Numerical results

This chapter will deeply analyze the performance of F-GBQ-PPO proposed in this paper. Facing to a one-to-one tracking problem, each function in the algorithm will be analyzed respectively. Taking the conventional PPO2(denote as PPO later) as the comparison benchmark, the overall simulation can be divided into three parts. The first part compares GBQ-PPO and PPO as a whole. The second part discusses the combination of guided policy, the introduction of hybrid exploration policy and the update of external parameters in GBQ-PPO; and the second part is the analysis of F-GBQ-PPO, which introduced the few-shot technique, on reducing the real sample demand.

**Figure 8:**
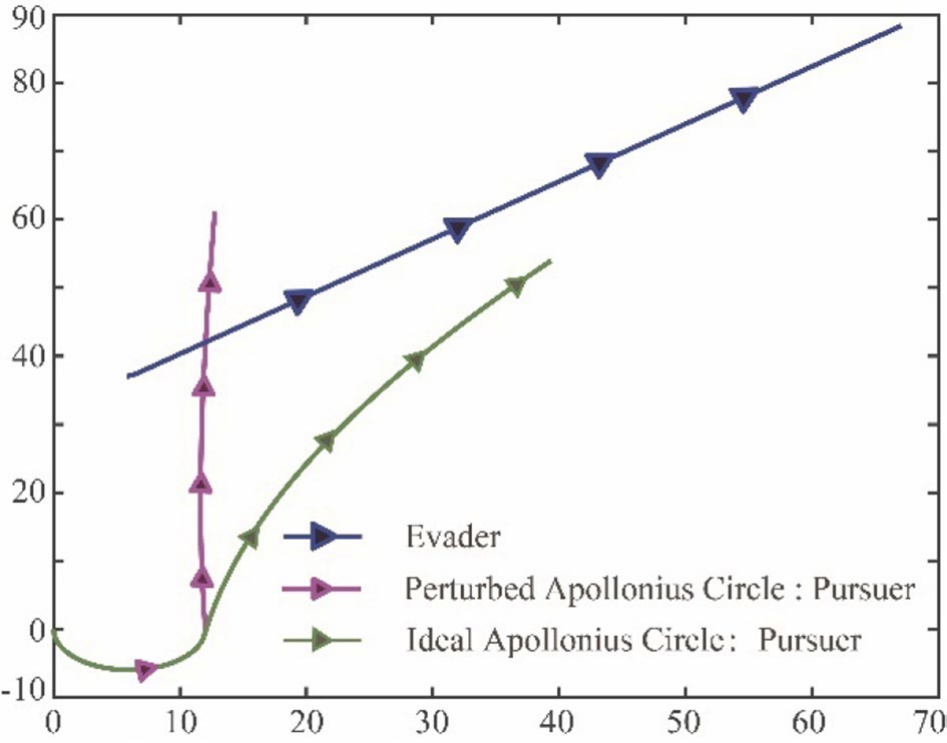
The traces of the pursuer under the policies from perturbed and ideal Apollonius circles.

It is supposed that there is one pursuer and one evader in the planar tracking problem, which can be detailly seen in Appendix. The initial position of *P* is $[0;0]$(*m*) with the heading angle of ${\theta }_{P}=-0.5\pi$ ($rad$), while that of *E* is $[\mathrm{5.78,37.01}]$(*m*) with the heading angle of ${\theta }_{E}=0.7$(*rad*). The maximum steering angle for the pursuer should not exist 0.5(*rad/s*). The velocities for the pursuer and the evader are 0.8*m/s* with the wheelbases of 3(*m/rad*) and 0.3(*m/rad*) respectively. To distinguish the real environment and the estimated model, the moving settings of evader are different. For the real environment, the evader follows the movement described in Eq. (44). Besides, in the estimated model, the estimated velocity error for the evader has -0.5 unit deviation in *x* direction and 0.8 unit deviation in *y* direction. Therefore, the policy obtained from the Apollonius circle based on the estimated model can be regarded as a perturbed one, and the agent which adopts it will deviate from the ideal condition to an extent. Figure 8 illustrates the traces of the pursuer under the policy from perturbed and ideal Apollonius circles in the applied environment, and the results can provide the reference baselines for the following simulations.

It is seen that the pursuer with a perturbed Apollonius circle has a roughly trend to track the evader, but due to the perturbation, the tracking effect is not good enough. Besides, the trace under ideal Apollonius circle shows that the pursuer cannot perfectly catch the evader but can only get as close to it as possible. Under the above scenario settings, the simulation results are as follows.

### Case 1: The overall performances between GBQ-PPO and PPO

**Figure 9:**
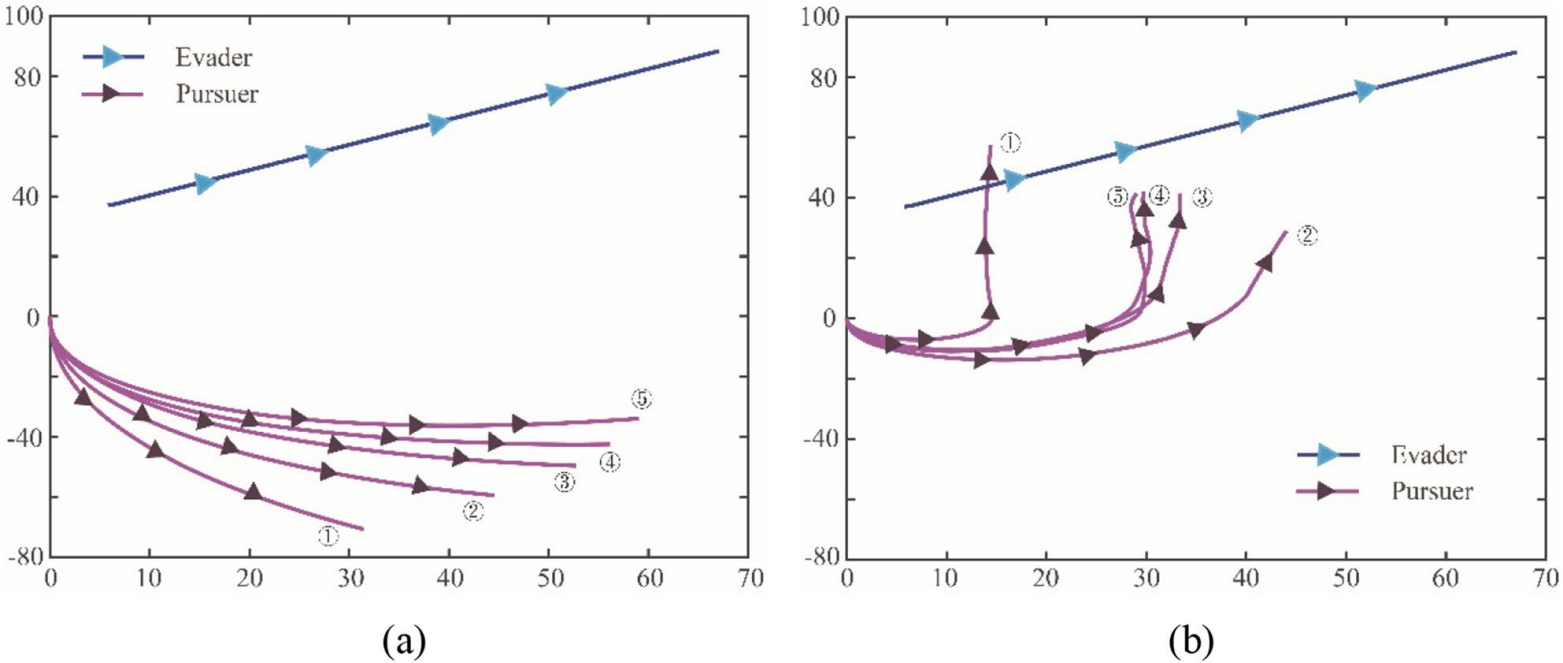
The five recorded traces of the pursuer under GBQ-PPO and PPO. (**a**) Agent adopts PPO (**b**) Agent adopts GBQ-PPO.

In the first case, it mainly compares the overall performance of GBQ-PPO and PPO. It is set that the parameters $m$ and $q$ in the external loop are updated every 20 episodes. Set the learning rate as $1{0}^{-3}$, the minibatch as 100, Fig. 9a and b show the traces of the pursuer and the evader after every 20 episodes update, respectively.

It can be seen from Fig. 9a that in normal PPO, the tracking effect is gradually and steadily improved with the increase of the number of episodes. After recording five times, which means 100 episodes have been conducted, compared with the initial downward motion trend, the trend of the pursuer has been adjusted to a certain degree according to the target. As can be seen in Fig. 9b, based on the guided policy generated by Apollonius circle, the pursuer shows a strong turning tendency at the first record. This is due to the judgment of the pursuer that it is better to follow the guided policy at this time. However, in the second record, it can be seen that there is a great difference between it and the first record. This is because with the update of the policy, after QGA optimization, the pursuer judges that it should reduce the dependence on the guided policy and adopt the RL policy to a greater extent. It can also be seen from the third, fourth and fifth records that, with the increasing number of episodes and QGA updates, the gap between adjacent recorded traces will also decrease. This is due to the fact that at the later stage of the iteration, the guided policy is no longer needed. In general, by comparing Fig. 9a and b, it can be seen that GBQ-PPO can apply the guided policy at the initial stage of learning, and gradually transition to use the RL policy at the later stage. Obviously, compared with the normal PPO algorithm, GBQ-PPO can significantly improve the learning effect.

**Figure 10:**
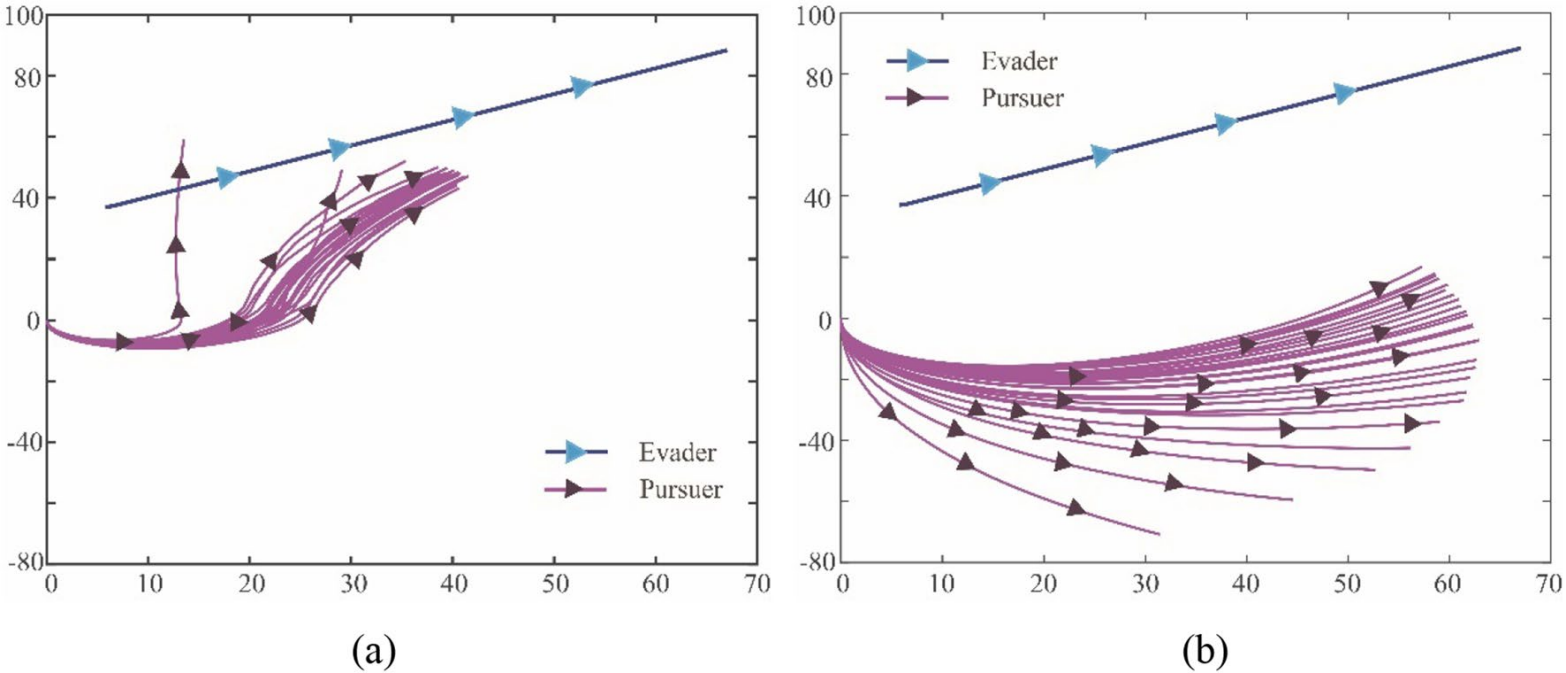
The twenty recorded traces of the pursuer under GBQ-PPO and PPO. (**a**) Agent adopts GBQ-PPO, (**b**) Agent adopts PPO.

**Figure 11:**
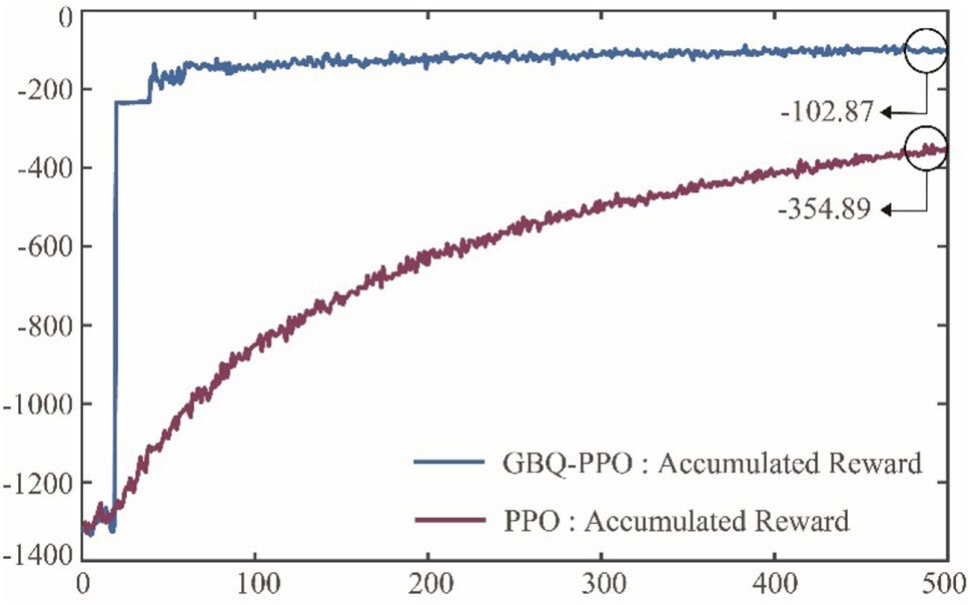
The accumulated rewards that the pursuer obtained under GBQ-PPO and PPO.

Figure 10a and b further show the learning results under GBQ-PPO and PPO. It can be seen from the figures that the learning effect of GBQ-PPO is obviously better than that of PPO at the same learning times, and its learning convergence trend is seeing to be shown. Figure 11 shows the change of accumulated rewards corresponding to Fig. 10a and b. It can be seen from Fig. 11 that with the increasing number of episodes, PPO will gradually increase the returned reward. After 500 episodes, it can reach a value of about -354.89 (an average of 10 times). In GBQ-PPO, after the first QGA update, the reward has been improved in a vertical-rise like manner, and the further optimization is carried out on this basis. After 500 episodes, it can reach a value of about -102.87, which is about 71% higher than the normal PPO.

### Case 2: the module performances in GBQ-PPO

**Figure 12:**
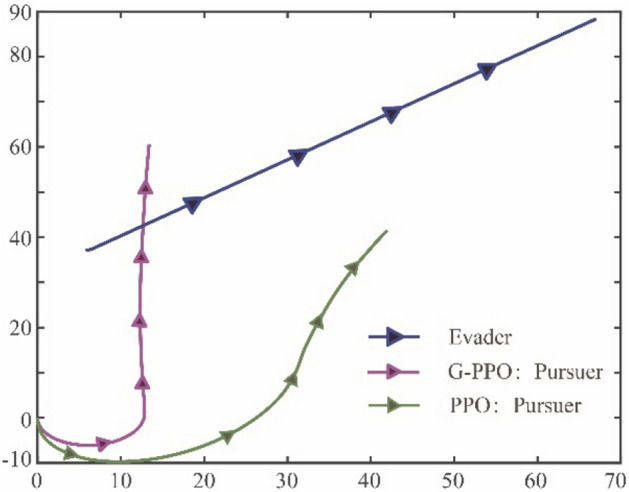
The traces of the pursuer under G-PPO and PPO after 1100 episodes.

**Figure 13:**
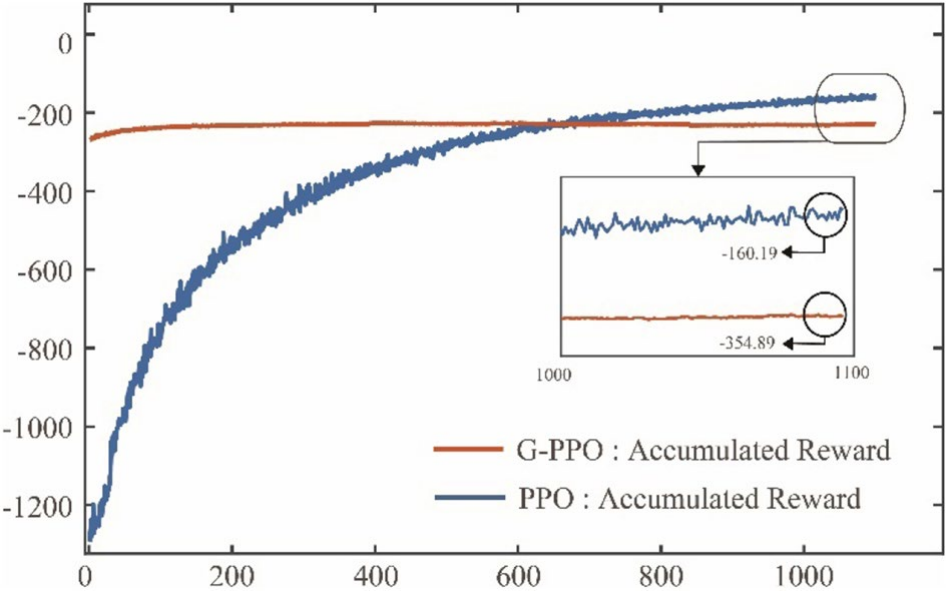
The accumulated rewards that the pursuer obtained under G-PPO and PPO.

This section mainly explains the roles of the modules in GBQ-PPO. More specifically, the three new modules in GBQ-PPO include: (1) guided policy based on Apollonius circle, (2) hybrid exploration policy based on biological motions, and (3) update of external parameters based on QGA. In the following, the actual performance of each module will be gradually analyzed. Here, it is denoted that the algorithm that only introduces the guided policy into PPO as G-PPO (the ratio parameter $m$ of the guided policy is set as 0.5), and it is compared with PPO after 1100 episodes. The results are shown in Figs. 12 and 13.

It can be seen from Fig. 12 that the training effect of G-PPO is not good enough, which is similar to the result of the first trace record in Fig. 9b. This shows that after the agent being assisted by the guided policy at the initial stage, the subsequent improvement effect is not obvious. This is mainly because that the ratio of the guided policy is fixed. In the later training period, especially when the guided policy is not accurate, continuing to maintain a high proportion of the guided policy will hinder the further optimization of the agent. Therefore, after enough training episodes, the performance of PPO is better than that of G-PPO. This conclusion is also shown in Fig. 13, which draws the accumulated rewards under the G-PPO and PPO algorithms. It can be seen from the figure that in the early stage of training (before about 600 episodes), G-PPO has better tracking effect than PPO due to the role of guided policy, but in the later stage of training, with the increasing number of episodes, the performance of PPO will gradually exceed G-PPO (finally reach about − 160.19 and − 354.89 respectively). The result shows a warning that if we just simply add a guided policy, the agent may gain some advantages in the early stage, but it may constrain the optimization in the later stage of learning. Therefore, in order to overcome this shortcoming, the update of the ratio parameter $m$ will be need, which will employ QGA to find an appropriate parameter for the agent.

**Figure 14:**
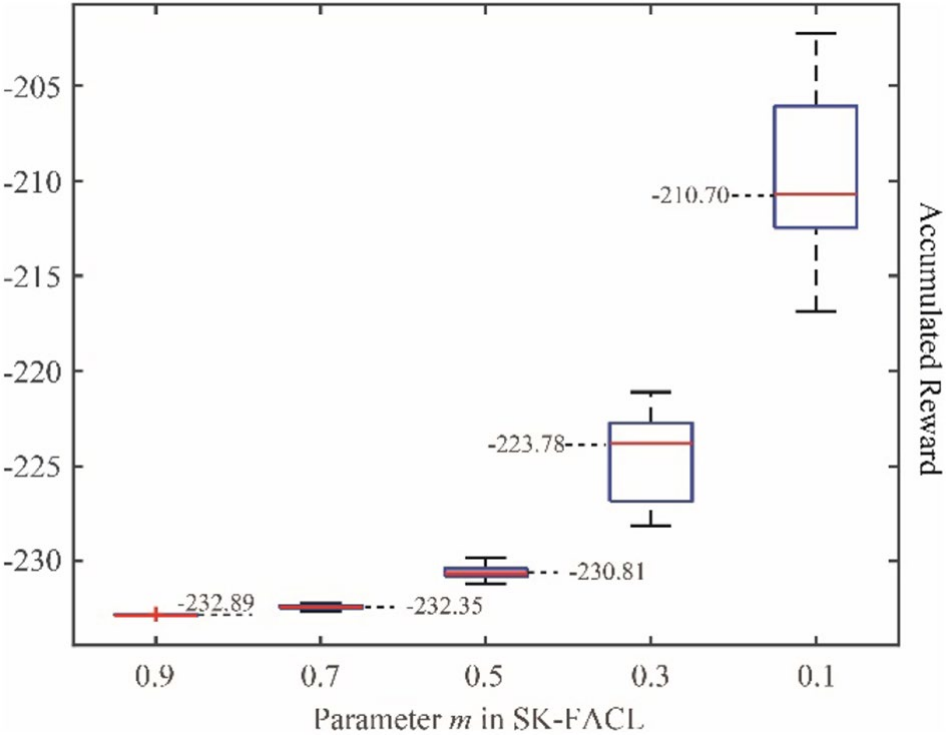
The accumulated rewards that the pursuer obtained under SK-FACL with different *m.*

The concept of G-PPO is a primary version of interpretable reinforcement learning algorithm, which is similar to SK-FACL proposed in^41^. SK-FACL is based on a fuzzy Actor-Critic learning framework where a guided policy is employed. To show the influences effected by the parameter *m*, we transplant the G-PPO to SK-FACL with different values of *m*. The results of accumulated rewards after 2000 episodes are shown in Fig. 14.

It is seen from the figure that the smaller value of *m,* the higher rewards will the pursuer obtain. This result indicates that the less guided policy we use, the better training result will finally get. However, if the guided policy is not used enough, the training efficiency would be unsatisfactory, which is already found in Fig. 13. Therefore, when apply G-PPO, it needs to consider how to make a compromise between the training effect and the training efficiency. To achieve this goal, an external loop is proposed to update the value of *m* fitting the training better.

**Figure 15:**
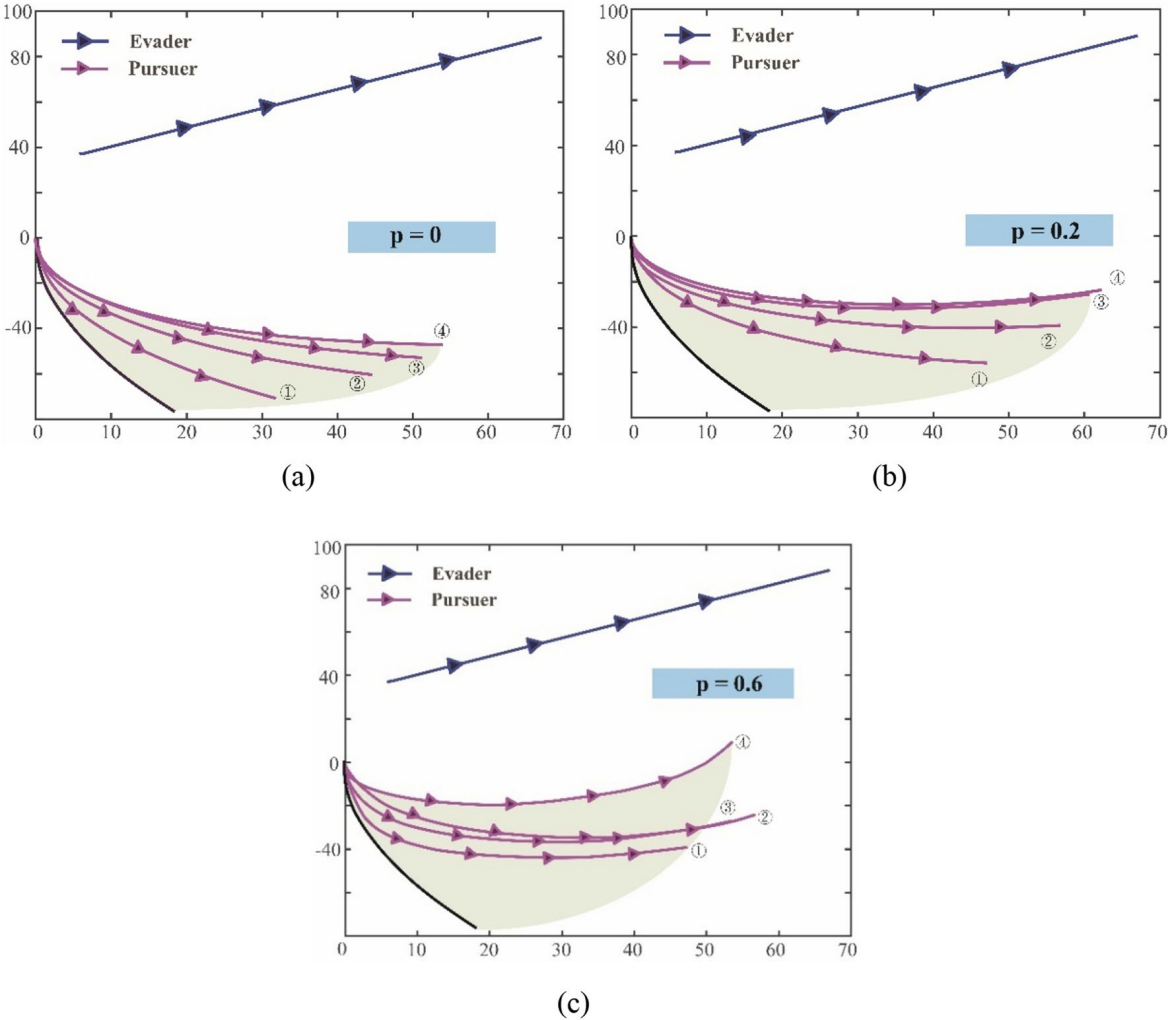
The coverage areas of the pursuer exploration after five records with different hybrid parameters. (**a**) The hybrid parameter $p=0$ (**b**) The hybrid parameter $p=0.2$, (**c**) The hybrid parameter $p=0.6$

For the performance of hybrid exploration policy based on biological motions, different values of $p$ (hybrid parameter) are selected for comparison. The $p$ reflects the proportion between Levy and Brown motions in the exploration policy, and the higher the $p$, the more effect of the Levy-motion. Therefore, it is reasonable that different selections of $p$ will bring different results. Figure 15a–c show coverage areas of the pursuer exploration after five records (20 episodes for each record) when $p=0$, $p=0.2$ and $p=0.6$, respectively.

The areas marked in the Fig. 15a–c are formed by the explorations of the pursuer, the number in the figure points out the trace recorded after every 20 episodes update, and the black-solid line represents the starting state of training as a benchmark. As $p=0$ in Fig. 15a, the pursuer completely adopts Brown exploration at this time. After four records, the area swept by its exploration traces constitutes the shadow area. Under the same number of explorations, it can be seen from Fig. 15a and b that the shadow area swept by the agent in Fig. 15b is larger than that in Fig. 15a, and the shadow coverage in Fig. 15c is larger than that in Fig. 15b. This shows that when the value of $p$ is higher, that is, when the exploration policy is closer to Levy motion, the exploration ability of the agent will be stronger. Under the same conditions, the agent can find favorable moving traces more quickly.

**Figure 16:**
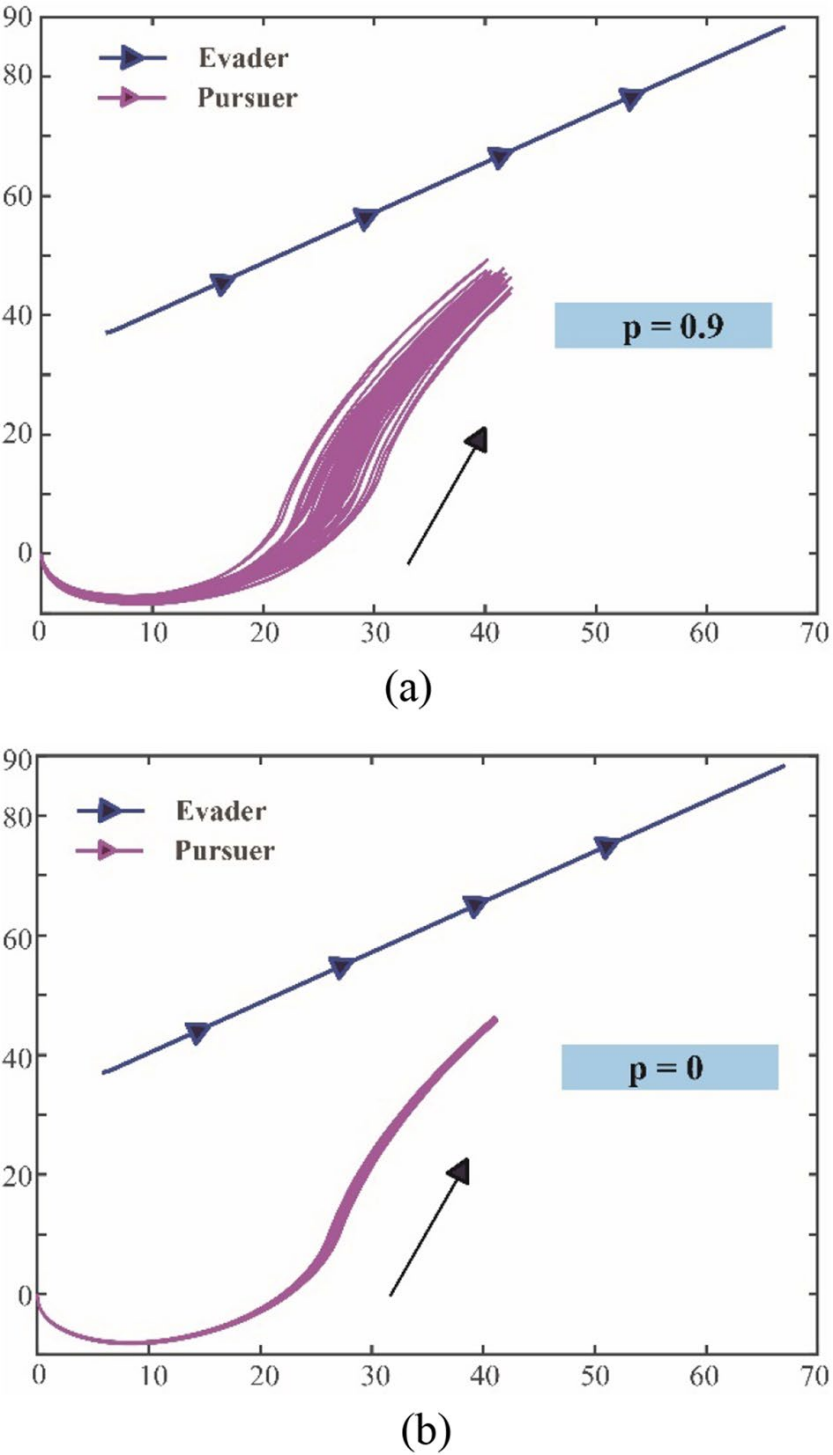
The fifty traces of the pursuer after learning convergence with different hybrid parameters. (**a**) The hybrid parameter $p=0.9$, (**b**) The hybrid parameter $p=0$.

However, it is not that the higher value of $p$, the better. Due to the large step size of Levy motion, it will be difficult to converge in the later stage of learning. Figure 16a and b show the conditions of the trace coverage of 50 times after learning convergence under the $p=0.9$ and $p=0$ respectively.

Compared with Fig. 16a and b, it can be clearly found that with a high proportion of Levy motion, the coverage area of traces after convergence will be large. It means that the agent will result in an inaccurate policy. At the same time, if the agent completely relies on Brown motion, the area of traces coverage after learning is much smaller, which indicates that the policy obtained is more accurate. These results show us that the hybrid parameter $p$ should be at a reasonable level. Ideally, more active large step length can be adopted in the early stage of learning, while in the later stage of learning, the step length can be appropriately shortened to adjust the policy parameters more finely. To achieve this goal, hybrid parameter $p$ is set as an external parameter and optimized through QGA. Benefited from the adjustment of $p$ along with episode process, the overall tracking performance can be optimized.

**Figure 17:**
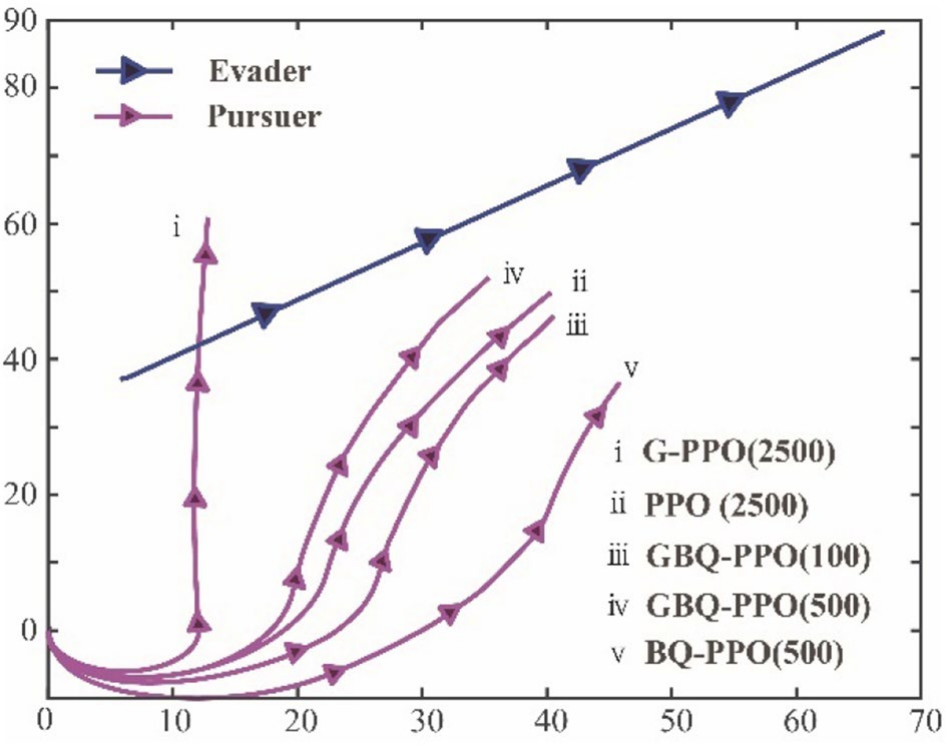
The traces of the pursuer under G-PPO, PPO, GBQ-PPO and BQ-PPO after different episode times.

The third module of GBQ-PPO is the update of external parameters based on QGA. This part is not independent, but combined with the aforementioned guided policy and hybrid exploration policy to optimize the external parameters $m$ and $q$. Figure 17 shows the traces of the pursuer under different algorithms of G-PPO, PPO GBQ-PPO and BQ-PPO.

**Figure 18:**
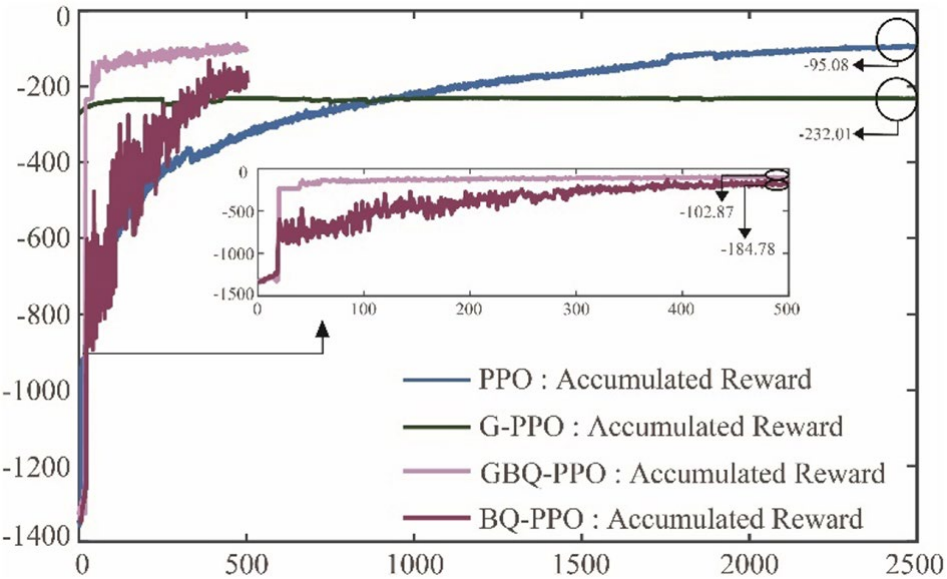
The total rewards that the pursuer obtained under G-PPO, PPO, GBQ-PPO and BQ-PPO.

Among them, G-PPO and PPO are the results after 2500 episodes. It can be seen that after enough episode’s update, PPO can achieve a good tracking effect, but G-PPO does not perform well at last. This result is also consistent with that in Fig. 13. Besides, Fig. 17 also shows the traces with GBQ-PPO after 100 and 500 episodes, respectively. It is seen that in GBQ-PPO, the pursuer can perform quite well only after 100 episodes. And when after 500 episodes, the performance is considerably close to that after 2500 episodes in PPO. Figure 18 shows the changes of the accumulated rewards of the three algorithms. It shows that G-PPO performs well in the early stage, but the optimization in the later period of learning is weak and ended by -232.01. Besides, PPO can steadily optimize the policy, reaching about -95.08 after 2500 episodes. In addition, GBQ-PPO can perfectly inherit the early-stage advantages of G-PPO, while continuing to maintain the capability of efficient policy optimization. Obviously, compared with PPO, GBQ-PPO greatly saves the number of episodes required but maintains the good performance. Considering a condition that if the guided policy is removed, only the hybrid parameter $p$ referring to the proportion of Levy motion can be updated, the algorithm is defined as BQ-PPO, and its performances are also shown in Figs. 17 and 18. It can be seen from the two figures that BQ-PPO still performs better than normal PPO. However, due to the lack of guided policy, it is inferior to GBQ-PPO, with the final reward at -184.78. It can be seen that the use and the update of external parameters based on QGA can effectively promote the efficient learning of agents. And when the external parameters of $m$ and $p$ participate in the update at the same time, the effect is the best.

### Case 3: The performance of F-GBQ-PPO

**Figure 19:**
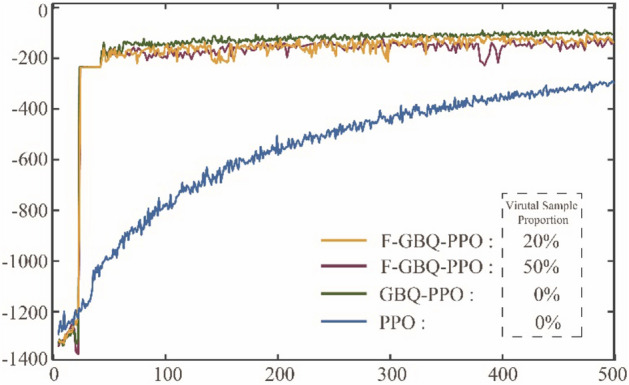
The accumulated rewards that the pursuer obtained under F-GBQ-PPO, GBQ-PPO and PPO.

In addition to GBQ-PPO, which improves PPO in three aspects, this paper also adds a function of state-prediction based on a multi-dimensional Gaussian process to realize the few-shot technique. In this way, the algorithm is newly denoted as F-GBQ-PPO, and its main goal is to provide fake samples so as to reduce the demand for real samples. Although the prediction based on Gaussian process is convenient, the generated fake samples will inevitably have errors with the real ones. In order to test whether these errors may affect the agent's decision-making, this case will analyze the performances of F-GBQ-PPO with different fake sample proportions. Since the generation of fake samples depends on the dataset composed of real samples, the use of fake samples shall not exceed 50%. Figure 19 shows the change of accumulated rewards with 20% and 50% of the fake samples respectively.

It can be seen from the figure that there is little difference in performance between F-GBQ-PPO with 20% of fake samples and 50% of fake samples. Besides, the performances of the two algorithms are close to that of GBQ-PPO without fake samples, and significantly better than that of PPO. These results show that the few-shot technique based on multi-dimensional Gaussian process has good application potential. When using a certain proportion of fake samples, it does not affect the overall learning of the agent, and can save a considerable number of real samples. Of course, it is inevitable that there must be errors between fake samples and real ones, which will affect the learning to some extent. As can be seen from Fig. 19, the rewards of the two F-GBQ-PPOs are slightly lower than that of GBQ-PPO. The specific accumulated reward values of these algorithms are shown in Table 1.

**Table 1:** The final mean values of accumulated rewards under F-GBQ-PPO, GBQ-PPO and PPO.

Name	Reward
F-GBQ-PPO (20%)	− 126.29
F-GBQ-PPO (50%)	− 138.56
GBQ-PPO	− 102.87
PPO	− 354.89

It can be seen from the table that F-GBQ-PPO with a certain proportion of fake samples has a slightly lower value of reward than GBQ-PPO, but still has significant advantages compared with normal PPO. Therefore, if the degree of learning effect deterioration is acceptable within a certain range, F-GQB-PPO algorithm can effectively save real samples, where saving 20% for F-GBQ-PPO (20%) and saving 50% for F-GBQ-PPO (50%), respectively.

To indicate the influences of hyper parameters in QGA, the experiments about F-GBQ-PPO, GBQ-PPO and BQ-PPO are conducted to show the performances. The concerned parameters including the permitted maximum generation and the size of population, denoted as “Maxgen” and “Sizepop” respectively. The results are drawn in Table 2.

**Table 2:** The accumulated rewards under F-GBQ-PPO, GBQ-PPO and BQ-PPO with different hyper parameters in QGA.

		F-GBQ-PPO(20%)	GBQ-PPO	BQ-PPO
Maxgen (Sizepop = 20)	2	− 207.27	− 96.61	− 96.97
	5	− 132.19	− 94.87	− 74.84
	10	− 80.87	− 73.37	− 82.72
Sizepop (Maxgen = 10)	5	− 84.56	− 82.48	− 78.05
	10	− 84.75	− 80.40	− 72.12
	20	− 80.87	− 73.37	− 82.72

**Figure 20:**
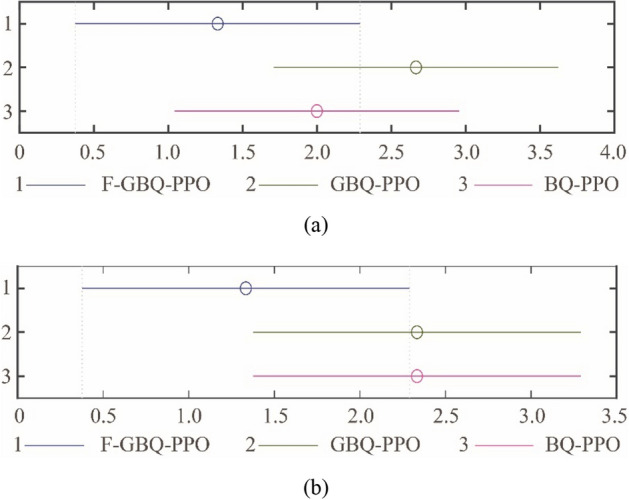
The mean ranks under F-GBQ-PPO, GBQ-PPO and PPO with different hyper parameters in QGA. (**a**) The mean ranks with different selections of Maxgen, (**b**) The mean ranks with different selections of Sizepop.

From the table, it is seen that the selection of Maxgen affects the three algorithms more that of Sizepop. In general, the performance of F-GBQ-PPO is worst because of the fake samples. It is obvious that the less of generation times, the fewer accumulated reward that F-GBQ-PPO will get. To further analyze the experimental results, the Friedman’s test is applied, the mean ranks are shown in Fig. 20a and b.

From Fig. 20a, it can be concluded that the performance of GBQ-PPO is the best and that of F-GBQ-PPO is the opposite, which means that the selection of Maxgen can distinguish the three algorithms well. However, in Fig. 20b, the performance of F-GBQ-PPO is still the worst, but the performances of GBQ-PPO and BQ-PPO are nearly the same. It says that the selection of Sizepop cannot differ the GBQ-PPO and BQ-PPO much. Therefore, from the experiments, we see that the selection of Maxgen in QGA may affect more on the proposed algorithm.

## Conclusion

To deal with the problem of insufficient interpretability in general reinforcement learning decisions, a multiple-interpretable improved PPO algorithm with few-shot technique is proposed and verified in a planar tracking problem in this paper. It improves the perceptual and logical interpretability by introducing the guided policy based on Apollonius circle and adding the hybrid exploration policy based on biological motions. In addition, the algorithm also forms an update loop of external parameter by encoding the external parameters and updating them based on QGA, thus improving the mathematical interpretability. In the simulated environment of a planar tracking scenario, the GBQ-PPO algorithm, which combines with the above improved modules, has been proved to have the ability to improve the learning efficiency greatly compared with normal PPO. Moreover, when the three modules work at the same time, the improvement effect is the best. On the basis of GBQ-PPO, a few- shot technique based on multi-dimensional Gaussian process is added to form F-GBQ-PPO to save the consumption of real samples. The experimental results show that the F-GBQ-PPO has the potential to effectively reduce the demand for real samples.

## Methods

We confirm that all methods were carried out in accordance with relevant guidelines and regulations and all experimental protocols were approved from Beijing University of Chemical Technology, Intelligent Science & Technology Academy Limited of CASIC, Key Lab of Aerospace Defense Intelligent System and Technology and Academy of Military Sciences.

## Figures and Tables

**Figure 21:**
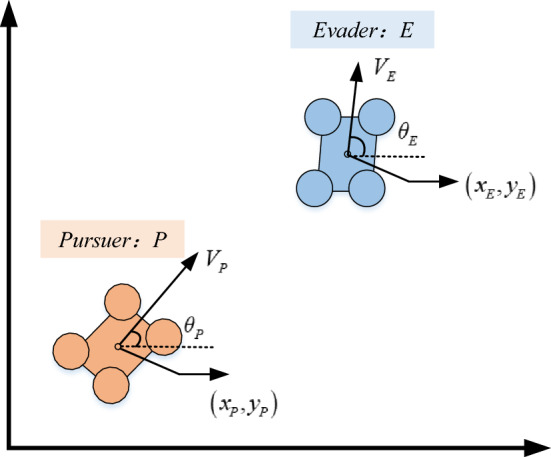
The diagram of plane tracking model.

## Data Availability

The datasets used and/or analysed during the current study available from the corresponding author on reasonable request.

## Appendix: The description of planar tracking problem

For the planar tracking problem considered in this paper, it is supposed that there exists two moving participants, including a pursuer and an evader. The agents move in the planar ground with constant speeds. The tacking model is drawn in Fig. 21.

The kinematic equation of the agent can be described as

44 $$\left\{\begin{array}{c}{\dot{x}}_{i}={v}_{i}\mathit{cos}\left({\theta }_{i}\right)\\ {\dot{y}}_{i}={v}_{i}\mathit{sin}\left({\theta }_{i}\right)\\ {\dot{\theta }}_{i}=\frac{{v}_{i}}{{L}_{i}}\mathit{tan}\left({u}_{i}\right)\end{array}\right.$$

where $i(i=P,E)$ represents the pursuer $P$ or the evader $E$, ${v}_{i}$ represents the value of constant speed, ${\theta }_{i}$ is the orientation, $({x}_{i},{y}_{i})$ is the position of the agent, ${L}_{i}$ represents the wheelbase, and ${u}_{i}$ represents the agent's steering angle, satisfying ${u}_{i}\in [-{u}_{imax},{u}_{imax}]$.

To optimize the policy of the agent based on a reinforcement learning process, the state should be maintained. Thus, in this tracking problem, the state is ${s}_{t}=\left[{\delta }_{p},\stackrel{.}{{\delta }_{p}}\right]$, where ${\delta }_{p}$ represents the angle difference between the direction of robot and the line-of-sight (LoS) to the other robot. The expressions of ${\delta }_{p}$ and $\stackrel{.}{{\delta }_{p}}$ are shown as below.

45 $${\delta }_{p}={{\text{tan}}}^{-1}(\frac{{y}_{e}-{y}_{p}}{{x}_{e}-{x}_{p}})-{\theta }_{p}={\text{cot}}(\frac{{y}_{e}-{y}_{p}}{{x}_{e}-{x}_{p}})-{\theta }_{p}$$

46 $${\dot{\delta }}_{p}=\frac{-1}{{{\text{sin}}}^{2}(\frac{{y}_{e}-{y}_{p}}{{x}_{e}-{x}_{p}})}\cdot \frac{({\dot{y}}_{e}-{\dot{y}}_{p})({x}_{e}-{x}_{p})-({y}_{e}-{y}_{p})({\dot{x}}_{e}-{\dot{x}}_{p})}{{({x}_{e}-{x}_{p})}^{2}}-\frac{{v}_{P}}{{L}_{P}}{\text{tan}}({u}_{p})$$

It is seen as a success if the pursuer can capture the evader when distance between them is less than a radius ${d}_{c}$, and the distance $d$ between the two agents can be obtained by follows.

47 $$d=\sqrt{{({x}_{e}-{x}_{p})}^{2}+{({y}_{e}-{y}_{p})}^{2}}$$

The reward function of the pursuer can be designed based on the change of the relative distance between the two agents. In principle, when the pursuer takes an action to get the distance be shorten, it should get a positive reward; otherwise, it should get a negative or lower reward.
